# Health-Related Physical Fitness in Patients With Inflammatory Bowel Disease vs Healthy Control Subjects

**DOI:** 10.1093/ibd/izaf169

**Published:** 2025-10-08

**Authors:** Karlijn Demers, Noortje van den Bergh, Bart C Bongers, Sander M J van Kuijk, Zlatan Mujagic, Daisy M A E Jonkers, Marieke J Pierik, Laurents P S Stassen

**Affiliations:** Department of Surgery, Maastricht University Medical Center+, 6229 HX Maastricht, the Netherlands; Department of Gastroenterology-Hepatology, Maastricht University Medical Center+, 6229 HX Maastricht, the Netherlands; Department of Surgery, Institute of Nutrition and Translational Research in Metabolism, Maastricht University, PO Box 616, 6200 MD Maastricht, the Netherlands; Department of Gastroenterology-Hepatology, Institute of Nutrition and Translational Research in Metabolism, Maastricht University, Maastricht, PO Box 616, 6200 MD, the Netherlands; Department of Gastroenterology-Hepatology, Maastricht University Medical Center+, 6229 HX Maastricht, the Netherlands; Department of Surgery, Institute of Nutrition and Translational Research in Metabolism, Maastricht University, PO Box 616, 6200 MD Maastricht, the Netherlands; Department of Nutrition and Movement Sciences, Institute of Nutrition and Translational Research in Metabolism, Maastricht University, PO Box 616, 6200 MD Maastricht, the Netherlands; Department of Clinical Epidemiology and Medical Technology Assessment, Maastricht University Medical Center, PO Box 5800, 6202 AZ Maastricht, the Netherlands; Department of Gastroenterology-Hepatology, Maastricht University Medical Center+, 6229 HX Maastricht, the Netherlands; Department of Gastroenterology-Hepatology, Institute of Nutrition and Translational Research in Metabolism, Maastricht University, Maastricht, PO Box 616, 6200 MD, the Netherlands; Department of Gastroenterology-Hepatology, Institute of Nutrition and Translational Research in Metabolism, Maastricht University, Maastricht, PO Box 616, 6200 MD, the Netherlands; Department of Gastroenterology-Hepatology, Maastricht University Medical Center+, 6229 HX Maastricht, the Netherlands; Department of Gastroenterology-Hepatology, Institute of Nutrition and Translational Research in Metabolism, Maastricht University, Maastricht, PO Box 616, 6200 MD, the Netherlands; Department of Surgery, Maastricht University Medical Center+, 6229 HX Maastricht, the Netherlands; Department of Surgery, Institute of Nutrition and Translational Research in Metabolism, Maastricht University, PO Box 616, 6200 MD Maastricht, the Netherlands

## Abstract

**Background:**

Inflammatory bowel disease (IBD) may negatively affect health-related physical fitness. However, the development of interventions to improve health-related physical fitness and thereby disease outcomes is hindered by insufficient evidence. This study compared health-related physical fitness between patients with IBD and healthy control subjects, examined associations with disease and treatment characteristics, and explored patients’ perspectives.

**Methods:**

In this cross-sectional study, 105 patients with IBD and 102 age- and sex-matched healthy control subjects performed validated tests for body fat (4-site skinfold thickness), cardiorespiratory fitness (steep ramp test), muscular strength (steep ramp test, 60-second sit-to-stand test, hand-held dynamometry), muscular endurance (isokinetic dynamometry), and flexibility (sit-and-reach test). Data on disease and treatment characteristics, fatigue, physical activity, and patients’ perspectives were collected.

**Results:**

Patients with IBD had higher body fat (29.5% vs 26.9%; *P *=* .*012), lower steep ramp test performance (peak work rate 4.2 W/kg vs 4.8 W/kg; *P *<* .*001), fewer sit-to-stand repetitions (42 vs 47; *P *=* .*002), and reduced hamstring strength (3.0 N/kg vs 3.2 N/kg; *P *=* .*011) compared with healthy control subjects. This was associated with higher age, female sex, higher body mass index, fatigue, arthritis, and multiple biologicals used. Most patients considered physical fitness important and beneficial for their symptoms, and the majority expressed interest in professional support.

**Conclusions:**

Patients with IBD have higher body fat and reduced cardiorespiratory fitness and muscular strength compared with healthy control subjects. Especially, patients with a higher age, female sex, higher body mass index, fatigue, arthritis, or multiple biologicals used are at risk for such impairments and may benefit from physical exercise interventions.

Key Messages *What is already known?*

Patients with inflammatory bowel disease (IBD) may exhibit reduced physical fitness, yet comprehensive assessments across all 5 health-related physical fitness components are lacking.

*What is new here?*

This is the first study to comprehensively assess all health-related physical fitness components in IBD, demonstrating higher body fat, lower cardiorespiratory fitness, and reduced muscular strength compared with matched control subjects, with these deficits associated with clinical and treatment-related factors. Moreover, most patients valued physical fitness and expressed a need for professional support.

*How can this study help patient care?*

These findings highlight the need for targeted physical exercise interventions to address physical fitness deficits in patients with IBD, which may complement current therapeutic strategies and potentially improve patient outcomes.

## Introduction

Inflammatory bowel disease (IBD), encompassing Crohn’s disease (CD) and ulcerative colitis (UC), is a chronic relapsing-­remitting inflammatory disorder of the gastrointestinal tract. While the disease primarily presents with gastrointestinal symptoms, it is frequently accompanied by systemic manifestations such as fatigue, joint pain, and diminished psychological and social well-being.^1^^,^^2^ Furthermore, disease-specific factors, including elevated levels of proinflammatory cytokines, malnutrition, diarrhea, medication-related side effects, and the need for surgical interventions, may lead to overall deconditioning and a decline in physical fitness.^3^^,^^4^ Moreover, it has been suggested that the substantial burden of symptoms associated with IBD reduce physical activity levels and increase sedentary behavior, likely further worsening physical fitness.^5^^,^^6^

Physical fitness is defined as the ability to carry out daily tasks with vigor and alertness, without undue fatigue, and with ample energy to enjoy leisure time pursuits and to meet unforeseen emergencies.^7^ A distinction can be made between skill- and health-related domains. Skill-related physical fitness encompasses performance attributes like agility, balance, and speed, whereas health-related physical fitness includes 5 core components, including body composition, cardiorespiratory fitness, muscular strength, muscular endurance, and flexibility. These components play an essential role in disease prevention and promoting long-term health outcomes.^8^ Diminished cardiorespiratory fitness and muscular strength are well-established prognostic indicators of chronic disease risk (eg, cardiovascular disease, type 2 diabetes, obesity) and overall mortality.^9^^,^^10^ Additionally, low cardiorespiratory fitness has been associated with adverse postoperative outcomes in abdominal surgery.^11^ Evidence suggests that targeted exercise-based interventions to improve health-related physical fitness reduce the risk of cardiovascular events and improve postoperative outcomes.^9^^,^^12^^,^^13^ In IBD, it is hypothesized that physical exercise improves subjective well-being and potentially even reduces disease activity.^14–18^

To date, information on health-related physical fitness in patients with IBD is limited.^19^ Identifying which health-related physical fitness components are compromised in patients with IBD and understanding how these deficits relate to disease-specific factors is essential for informing the development of targeted physical exercise training strategies that have the potential to improve disease outcomes and quality of life. Additionally, to design effective and sustainable interventions that align with patients’ needs, it is crucial to incorporate patients’ perspectives on physical fitness. Therefore, the primary aim of this study was to systematically assess differences in health-related physical fitness components between patients with IBD and age- and sex-matched healthy control subjects. Secondary aims were to investigate associations between health-related physical fitness components and disease- and treatment-related characteristics in patients with IBD and to explore patients’ perspectives on physical fitness.

## Methods

### Study design and participants

We conducted a cross-sectional study from August 2022 to September 2024 and included patients with IBD (CD and UC) and healthy control subjects. This study was part of a larger project addressing physical fitness in IBD and is registered at ClinicalTrials.gov under NCT05482932. Patients with IBD were consecutively recruited via the gastroenterology outpatient clinic of the Maastricht University Medical Center+. Eligible patients were those ≥18 years of age with a certified diagnosis of CD or UC, in remission or with mild-to-moderate clinical disease activity (Harvey-Bradshaw index ≤16 for CD or Simple Clinical Colitis Activity Index (SCCAI) ≤11 for UC). Healthy control subjects were recruited through IBD patients willing to participate, who were encouraged to invite a partner, family member, or friend, as well as through advertisements in public spaces (eg, supermarkets, hospital, university), social media platforms, and a local newspaper. Healthy control subjects had to be ≥18 years of age and without a diagnosis of CD or UC. Patients and healthy control subjects were required to have an American Society of Anesthesiologists physical status of ≤2, indicating that they were generally healthy or had only mild systemic comorbidities, aside from the presence of IBD in the patient group. Patients and healthy control subjects were excluded in case of any injuries, (neuro)muscular, rheumatic, or orthopedic comorbidities that could potentially affect study evaluations. Pregnant or lactating women and (competitive or elite) athletes were also excluded. Patients and healthy control subjects were matched for age and sex at the group level.

### Health-related physical fitness assessment

First, body mass and height of all participants were measured (barefoot and in light clothing) to calculate body mass index (BMI). Mid-upper arm circumference was measured at the posterior side of the nondominant arm. Next, participants performed various field-based tests for each of the components of health-related physical fitness. For this study, only tests demonstrating strong correlation or agreement with gold-standard measures based on previous validation (correlation coefficient >0.70) were selected.^20^ The test protocols have been described in more detail elsewhere.^20^ Body fat percentage was evaluated by skinfold thickness measurements at 4 sites (ie, biceps, triceps, subscapular, and suprailiac). Cardiorespiratory fitness and muscular strength were quantified by the steep ramp test, a short-term, maximal exercise test on a cycle ergometer. The work rate increased in a ramp-like manner until volitional exhaustion, recording the work rate at peak exercise (WR_peak_) as main outcome. Hamstring muscular strength was further assessed using the 60-second sit-to-stand test and hand-held dynamometry. Muscular endurance of the quadriceps and hamstring muscles was measured by calculating the peak torque fatigue index and the work fatigue index over 30 maximal repetitions performed on the Biodex System 4 Pro dynamometer. Strength and endurance measurements were performed on the dominant leg. Flexibility was assessed using the sit-and-reach test, requiring participants to reach forward 3 times from a seated position with extended legs against a measuring box, recording the greatest distance to the nearest centimeter.

### Fatigue assessment

Fatigue was assessed to investigate its association with health-related physical fitness, as it is a common and clinically significant symptom in patients with IBD. Investigating this association could provide more evidence for targeted physical exercise interventions aimed mitigating fatigue by improving health-related physical fitness in this population. The validated Dutch Checklist Individual Strength (CIS) was used to examine fatigue in all participants.^21^ It is a 20-item questionnaire assessing 4 dimensions of fatigue, including subjective fatigue, concentration, motivation, and activity levels over the past 2 weeks, with a higher score indicating more severe fatigue.

### Physical activity assessment

Physical activity was assessed to examine its association with health-related physical fitness deficits as a potential behavioral target for interventions, and to account for possible confounding effects in statistical analyses. Self-reported physical activity levels were evaluated using the Dutch-translated version of the International Physical Activity Questionnaire (IPAQ) short form.^22^ This questionnaire addresses the frequency and duration of physical activity across various intensities (walking, moderate, and vigorous intensity) over the past 7 days. Total and intensity-specific physical activity scores were calculated in metabolic equivalent of task minutes per week. The IPAQ short form also included a question on daily sitting time over the past 7 days, which was expressed in total minutes per day. Data processing and analysis was performed according to the official IPAQ scoring protocol.

### Assessment of patients’ perspectives on physical fitness

A questionnaire was developed to capture patients’ perspectives on physical fitness, in accordance with B1-level Dutch language guidelines ensuring accessibility for the majority of the Dutch population. This questionnaire included items on the relationship between (improving) physical fitness and intestinal symptoms, as well as symptoms of fatigue, insomnia, and depression. Additionally, patients were asked to score their perceived importance of physical fitness, and which barriers they encounter to (increase) physical activity or exercise. Further, they were asked about their willingness to improve physical fitness with professional support, if they currently receive support from the hospital, and if they would be interested in additional assistance.

### Demographic and clinical data collection

Demographic data of patients with IBD and healthy control subjects were collected during the study visit, including age, sex, educational level, and current employment status. Additionally, information about smoking status and indicators of malnutrition (ie, weight loss and food intake) were collected at inclusion. The presence of malnutrition was defined according to the Global Leadership Initiative on Malnutrition, requiring 1 positive phenotype criterium in combination with 1 etiologic criterium.^23^ The phenotype criteria included (1) nonvoluntary weight loss (>5% within the past 6 months or >10% beyond 6 months), (2) low BMI (<20 kg/m^2^ when <70 years of age and <22 kg/m^2^ when >70 years of age), or (3) low muscle mass (mid-upper arm circumference <24 cm).^24^ Etiologic criteria included (1) active inflammation, either clinical (HBI ≥5 or SCCAI ≥3) or biochemical (fecal calprotectin >250 µg/g), at inclusion; or (2) subjective reduced food intake or assimilation, including a prior intestinal resection. Participants were also asked about comorbidities according to the Charlson comorbidity index.^25^ For patients with IBD, comorbidities were verified through review of clinical records.

For all patients with IBD, the HBI and SCCAI were administered at inclusion to assess clinical disease activity. Clinical data were collected from electronic patient records, including comorbidities, disease entity, Montreal classification, disease duration, extraintestinal manifestations, medication use, total number of biologicals used, and prior intestinal resections. Fecal calprotectin values were collected if available within a 6-week period around inclusion, with values under 250 µg/g being indicative of biochemical remission. Additionally, IBD-Control questionnaires were collected from the MyIBDcoach telemonitoring platform if available within a 4-month period around inclusion.^26^ Briefly, MyIBDcoach is a platform used for disease monitoring in routine clinical care, with patients completing questionnaires at intervals ranging from 1 to 4 months, depending on disease activity and patient preference. The IBD-Control captures disease control from the patient’s perspective and consisted of a IBD-Control-8 score (range 0-16) and a IBD-Control visual analog scale score (range 0-100), with higher scores indicating better disease control.^27^

### Statistical analyses

Statistical analyses were performed using IBM SPSS Statistics 27.0. Demographic and clinical characteristics were summarized using descriptive statistics. Continuous variables were reported as mean ± SD or median (Q1-Q3), based on normality and the presence of outliers. Group differences were assessed using the independent samples *t* tests or the Mann-Whitney *U* test for numerical variables, and Pearson’s chi-square test or Fisher’s exact test for categorical variables. For the primary objective, physical fitness outcomes were compared between patients with IBD and healthy control subjects, with groups matched for age and sex. Additionally, multivariable linear regression analysis was conducted to adjust differences for demographic and clinical characteristics (ie, age, sex, Charlson comorbidity index, smoking status, and educational level) while investigating the differences in physical fitness outcomes between patients with IBD and healthy control subjects. Adjustments were limited to variables not influenced by IBD status to avoid overadjustment. Variables such as employment status, fatigue, and physical activity were not included in the models, as these are likely consequences of the disease, rather than independent confounders. To examine whether differences in physical fitness outcomes were attributable to active disease, a sensitivity analysis was performed in the subgroup of patients with IBD who were in biochemical remission (defined as fecal calprotectin <250 µg/g).

For the secondary objectives, univariable and multivariable linear regression analyses were performed to investigate associations of physical fitness test outcomes with patient-, disease-, and treatment-related characteristics in patients with IBD. Initially, univariable linear regression was used to assess the individual association between each patient-, disease-, or treatment-related characteristic and each of the physical fitness test outcomes. Variables with a *P* value <.10 were subsequently included in the multivariable model, followed by backward elimination on the Wald test, removing the variable with the highest *P* value until all remaining variables had a *P* value <.10. In cases of multicollinearity (variance inflation factor >5) between 2 (independent) variables, only the variable with the strongest correlation coefficient with the physical fitness test outcome (dependent variable) was retained, prioritizing age and sex over other variables. Finally, exploratory findings of patients’ perspectives on physical fitness were presented using descriptive statistics. A 2 -tailed *P* value <.05 was considered statistically significant.

### Ethical statement

This study was conducted in compliance with the Declaration of Helsinki and was approved by the Medical Ethical Committee of the Maastricht University Medical Center+ (registration no. 22-012). All patients gave written informed consent.

## Results

### Study population

Participants’ characteristics for patients with IBD (n = 105) and healthy control subjects (n = 102) are shown in Table 1. There were no statistically significant differences in age, sex, and comorbidities between the 2 groups. However, patients with IBD had a higher BMI (25.8 kg/m^2^ vs 24.4 kg/m^2^; *P *=* .*016) and malnutrition rate (5.7% vs 0%; *P = .*029) compared with healthy control subjects. Significant group differences were also found for educational level (*P *<* .*001), employment status (*P *=* .*005), and smoking status (*P *<* .*001). Numerically, patients with IBD were less likely to have a university-level education (16.2% vs 43.0%), were less frequently employed full-time (33.3% vs 53.0%), and were more often on sick leave or partially or fully unfit to work (16.2% vs 3.0%) as compared with healthy control subjects. Additionally, former smoking rates were higher among the IBD group (50.5% vs 26.5%). Furthermore, patients with IBD showed statistically significant higher fatigue scores (*P *<* .*001) and had lower metabolic equivalent of task minutes per week in vigorous-intensity physical activity compared with healthy control subjects (*P *=* .*002).

**Table 1.** Characteristics of patients with IBD and healthy control subjects.

	IBD patients (n = 105)	HC subjects (n = 102)	*P*
**Demographic and clinical characteristics**			
Age at inclusion, y	43.0 (30.0-56.6)	38.0 (26.2-60.8)	.703
Female	53 (50.5)	52 (51.0)	1.000
Charlson comorbidity index			.514
0	59 (56.2)	55 (53.9)	
1-2	36 (34.3)	32 (31.4)	
>2	10 (9.5)	15 (14.7)	
BMI, kg/m^2^	25.8 ± 4.1	24.4 ± 3.9	.016^*^
BMI			.309
Underweight (<18.5 kg/m^2^)	2 (1.9)	1 (1.0)	
Normal weight (18.5-24.9 kg/m^2^)	49 (46.7)	60 (58.8)	
Overweight (≥25.0 kg/m^2^)	39 (37.1)	32 (31.4)	
Obesity (≥30.0 kg/m^2^)	15 (14.3)	9 (8.8)	
Malnutrition	6 (5.7)	0 (0.0)	.029^*^
Education level^a^			<.001^*^
Primary education	0 (0.0)	0 (0)	
Secondary education	25 (23.8)	16 (16.0)	
Intermediate vocational education	38 (36.2)	15 (15.0)	
Higher vocational education	25 (23.8)	26 (26.0)	
University	17 (16.2)	43 (43.0)	
Employment status^a^			.005^*^
Working full-time	35 (33.3)	53 (53.0)	
Working part-time	27 (25.7)	14 (14.0)	
Studying	13 (12.4)	16 (16.0)	
Retired	13 (12.4)	14 (14.0)	
Sick leave	6 (5.7)	1 (1.0)	
Partially or fully unfit to work	9 (8.6)	1 (1.0)	
Not working for other reasons	2 (1.9)	1 (1.0)	
Smoking status			<.001^*^
Current smoker	12 (11.4)	11 (10.8)	
Former smoker	41 (39.1)	16 (15.7)	
Quit <6 mo ago^b^	39 (97.5)	0 (0)	
Quit ≥6 mo ago^b^	1 (2.5)	16 (15.7)	
Never smoked	52 (49.5)	75 (73.5)	
CIS total score^c^	63 (45-84)	43 (32-59)	<.001^*^
CIS subjective fatigue^c^	30 (20-40)	16.0 (12-24)	<.001^*^
CIS concentration^c^	13 (9-20)	11 (8-16)	.111
CIS motivation	9 (7-14)	8 (5-11)	<.001^*^
CIS activity	7 (5-11)	6 (4-9)	.052
IPAQ total, MET-min/wk^d^	3464 (2346-6560)	3639 (2346-6375)	.762
IPAQ walking, MET-min/wk^e^	1097 (693-2079)	990 (446-1386)	.136
IPAQ moderate intensity, MET-min/wk^f^	720 (340-1920)	960 (480-2160)	.357
IPAQ vigorous intensity, MET-min/wk^g^	480 (0-1920)	1440 (480-2400)	.002^*^
IPAQ sitting, min/d^h^	360 (240-480)	360 (240-540)	.717
**Disease characteristics**			
Disease entity			
CD	55 (52.4)		
UC	50 (47.6)		
Montreal age at diagnosis			
A1: ≤16 y	7 (6.7)		
A2: 17-40 y	75 (71.4)		
A3: >40 y	23 (21.9)		
Montreal disease location (CD)			
L1: ileal	18 (32.7)		
L2: colonic	12 (21.8)		
L3: ileocolonic	25 (45.5)		
+ Perianal disease	10 (18.2)		
+ Upper gastrointestinal disease	5 (9.1)		
Montreal disease behavior (CD)			
B1: nonstricturing, nonpenetrating	33 (60.0)		
B2: stricturing	9 (16.4)		
B3: penetrating	13 (23.6)		
Montreal disease extension (UC)			
E1: proctitis	5 (10.0)		
E2: left-sided colitis	17 (34.0)		
E3: pancolitis	28 (56.0)		
Disease duration, y	10.5 (5.4-20.8)		
HBI score (CD)	3 (2-5)		
SCCAI score (UC)	1 (0-2)		
Clinical disease activity^i^			
Remission	80 (76.2)		
Mild disease activity	24 (22.9)		
Moderate disease activity	1 (1.0)		
Fecal calprotectin, μg/g^j^	47 (17-201)		
Biochemical disease activity^j^			
Remission	80 (81.6)		
Active disease	18 (18.4)		
IBD-Control-8 score^k^	14 (10-16)		
IBD-Control-VAS score^k^	87 (70-97)		
EIM during disease course^l^			
≥1 EIM during disease course	52 (49.5)		
Uveitis or scleritis	8 (7.6)		
Primary sclerosing cholangitis	2 (1.9)		
Arthralgia	31 (29.5)		
Arthritis	18 (17.1)		
Axial	7 (6.7)		
Peripheral	13 (12.4)		
Current IBD medication			
None	19 (18.1)		
Mesalazine only	18 (17.1)		
(Topical) corticosteroids	6 (5.7)		
Immunomodulators	13 (12.4)		
Biologic agents	55 (524)		
Number of biologicals during disease course	1 (0-2)		
Prior intestinal resection	22 (21.0)		

Values are median (Q1-Q3), n (%), or mean ± SD.

Abbreviations: BMI, body mass index; CD, Crohn’s disease; CIS, Checklist Individual Strength; EIM, extraintestinal manifestation; HBI, Harvey-Bradshaw index; HC, healthy control; IBD, inflammatory bowel disease; IPAQ, International Physical Activity Questionnaire; MET, metabolic equivalent of task; SCCAI, Simple Clinical Colitis Activity Index; UC, ulcerative colitis; VAS, visual analog scale.

* Significant *P *< .05.

a n* = *2 missing in the HC group.

b n* = *1 missing in the IBD group.

c n* = *1 missing in the HC group.

d Available in n* = *92 in the IBD group and n* = *91 in the HC group, missing data due to responses of “I don’t know.”

e Available in n* = *96 in the IBD group and n* = *98 in the HC group, missing data due to responses of “I don’t know.”

f Available in n* = *102 in the IBD group and n* = *99 in the HC group, missing data due to responses of “I don’t know.”

g Available in n* = *101 in the IBD group and n* = *94 in the HC group, missing data due to responses of “I don’t know.”

h Available in n* = *98 in the IBD group and n* = *97 in the HC group, missing data due to responses of “I don’t know.”

i Clinical disease activity according to the HBI for CD and the SCCAI for UC; remission was defined as HBI < 5 or SCCAI < 3, mild disease activity as HBI 5-7 or SCCAI 3-5, and moderate disease activity as HBI 8-16 or SCCAI 6-11.

j Available in n* = *98 in the IBD group; remission was defined as fecal calprotectin <250 µg/g.

k Available in n* = *56 in the IBD group within 4 months around inclusion.

l Values represent the number of unique patients who experienced EIMs at least once during their disease course. Recurrent episodes of the same EIM in the same individual were not counted multiple times. A single patient may have had more than 1 type of EIM.

Of the patients with IBD, 55 (52.4%) were diagnosed with CD and 50 (47.6%) were diagnosed with UC (Table 1). The majority were in remission, with 76.2% in clinical remission and 81.6% in biochemical remission. Most patients (52.4%) were currently receiving biologic therapy. Characteristics for patients with CD and UC separately can be found in Table S1.

### Differences in health-related physical fitness between patients with IBD and healthy control subjects

**Figure 1.**
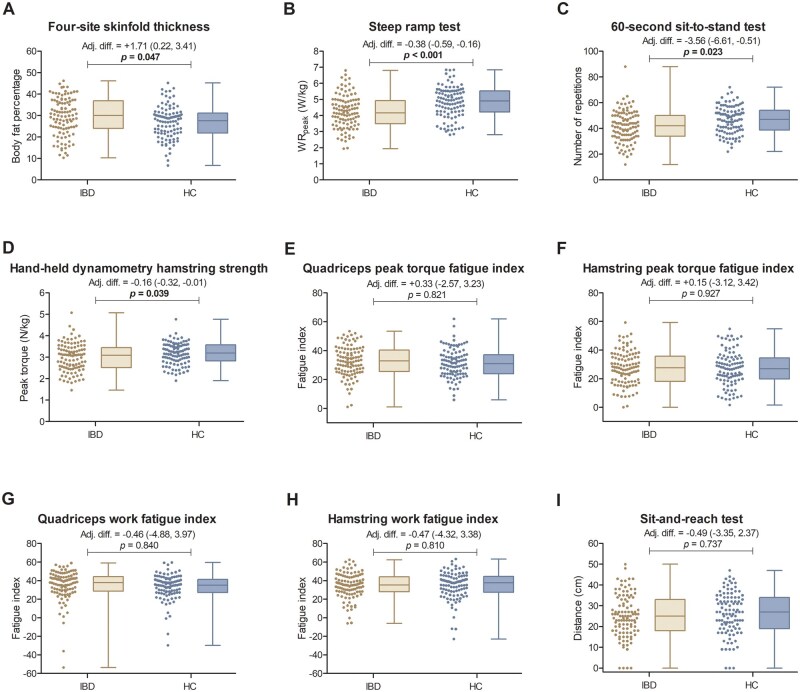
Unadjusted distributions and adjusted differences in health-related physical fitness outcomes between patients with inflammatory bowel disease (IBD) and healthy control subjects. Scatter and box-and-whisker plots show physical fitness test outcomes for patients with IBD (n = 105) and healthy control (HC) subjects (n = 102), with adjusted differences and corresponding 95% confidence intervals displayed above the plots. Adjusted differences were derived from multivariable linear regression analyses, adjusted for age, sex, Charlson Comorbidity Index, smoking status, and educational level. Scatter plots display individual data points. Box-and-whisker plots show the interquartile range (box), median (horizontal line), and minimum and maximum values (whiskers). Statistically significant differences (*P *< .05) are highlighted in bold. adj. diff., adjusted difference.

Differences in health-related physical fitness tests outcomes between patients with IBD and healthy control subjects are displayed in Figure 1, with a detailed overview of the adjusted analyses provided in Tables S2 and S3. Patients with IBD had significantly higher body fat percentage compared with healthy control subjects (29.5% vs 26.9%; adjusted difference +1.71, *P *=* .*047). The performance at the steep ramp test was significantly lower in patients with IBD than in healthy control subjects (WR_peak_ 4.2 W/kg vs 4.8 W/kg; adjusted difference −0.38, *P *<* .*001), indicating lower cardiorespiratory and muscular strength. Lower muscular strength in patients with IBD was also shown by a lower number of repetitions on the 60-second sit-to-stand test (42 vs 47; adjusted difference −3.56, *P *=* .*023) and lower hand-held dynamometry hamstring strength (3.0 N/kg vs 3.2 N/kg; adjusted difference −0.16, *P *=* .*039). In the sensitivity analyses limited to patients with IBD in biochemical remission, similar or even larger effect estimates were observed (Table S4). No significant differences were observed in muscular endurance or flexibility between patients with IBD (both in the total population and in those in biochemical remission) and healthy control subjects (Figure 1; Tables S2-S5).

Table S6 provides separate physical fitness test outcomes for patients with IBD, CD, and UC and for healthy control subjects, including exploratory between-group comparisons, with outcomes adjusted for demographic and clinical characteristics presented in Tables S7 to S10.

### Associations with health-related physical fitness in patients with IBD

Multivariable linear regression analyses among patients with IBD showed that higher age, female sex, and higher BMI were significantly associated with higher body fat percentage (Table 2). Further, higher age, female sex, higher BMI, and higher CIS subjective fatigue subscale scores were associated with lower WR_peak_ on the steep ramp test (Table 3), reflecting lower cardiorespiratory fitness and muscular strength. Similarly, higher age, female sex, and higher BMI were significantly associated with lower muscular strength reflected by fewer 60-second sit-to-stand repetitions and lower hand-held dynamometry hamstring strength. Additional factors associated with worse sit-to-stand performance were higher CIS motivation subscale scores, prior arthritis, and a greater number of biologicals used during the disease course. Furthermore, various factors were associated with reduced muscular endurance, reflected by higher fatigue indexes (Table 4). These included younger age, lower Charlson comorbidity index, not being employed, higher CIS subjective fatigue subscale scores, the presence of CD vs UC, and not using mesalazine medication only. Finally, male sex and higher BMI were significantly associated with poorer flexibility on the sit-and-reach test (Table 5).

**Table 2.** Univariable and multivariable linear regression analyses of the associations of patient, disease, and treatment characteristics with body fat percentage measured by 4-site skinfold thickness in patients with IBD.

	Univariable		Multivariable	
	*B* (95% CI)	*P*	*B* (95% CI)	*P*
**Patient characteristics**				
Age at inclusion	0.28 (0.18 to 0.37)	<.001^*^	0.24 (0.14 to 0.34)	<.001^*^
Male	−10.07 (−12.83 to −7.31)	<.001^*^	−9.58 (−11.26 to −7.91)	<.001^*^
Charlson comorbidity index	3.57 (2.07 to 5.06)	<.001^*^	−1.25 (−2.72 to 0.23)	.097
BMI	1.25 (0.92 to 1.59)	<.001^*^	1.02 (0.82 to 1.22)	<.001^*^
Malnutrition	0.62 (−6.68 to 7.92)	.867		
Smoking status				
Nonsmoker	REF	REF		
Current smoker	0.48 (−4.84 to 5.81)	.858		
Educational level				
Low/medium^a^	REF	REF		
High^b^	−1.65 (−5.09 to 1.80)	.344		
Employment status				
Nonemployed^c^	REF	REF		
Employed^d^	−4.68 (−8.01 to −1.36)	.006^*^		
CIS total score	0.06 (0.00 to 0.13)	.056		
CIS subjective fatigue	0.18 (0.05 to 0.31)	.006^*^		
CIS concentration	0.10 (−0.11 to 0.31)	.345		
CIS motivation	0.16 (−0.17 to 0.48)	.340		
CIS activity	0.01 (−0.40 to −0.43)	.948		
IPAQ total^e^	0.00 (0.00 to 0.00)	.078	0.00 (0.00 to 0.00)	.068
IPAQ walking^f^	0.00 (0.00 to 0.00)	.575		
IPAQ moderate intensity^g^	0.00 (0.00 to 0.00)	.245		
IPAQ vigorous intensity^h^	0.00 (0.00 to 0.00)	.013^*^		
IPAQ sitting^i^	0.01 (0.00 to 0.01)	.245		
**Disease characteristics**				
Disease entity				
CD	REF	REF		
UC	−1.54 (−4.92 to 1.84)	.369		
Disease duration	0.22 (0.08 to 0.37)	.003^*^	0.08 (−0.01 to 0.17)	.072
Clinical active disease^j^	3.00 (−0.93 to 6.94)	.133		
Fecal calprotectin^k^	0.00 (−0.01 to 0.00)	.139		
IBD-Control-8 score^l^	−0.06 (−0.68 to 0.56)	.851		
IBD-Control-VAS score^l^	−0.04 (−0.15 to 0.07)	.445		
Arthralgia during disease course	4.73 (1.14 to 8.33)	.010^*^		
Arthritis during disease course	1.56 (−2.93 to 6.04)	.493		
**Treatment characteristics**				
No current medication use	3.39 (−0.96 to 7.74)	.125		
Current use of mesalazine only	−0.11 (−4.61 to 4.38)	.961		
Current use of corticosteroids	−2.70 (−8.34 to 2.95)	.346		
Current use of immunomodulator	−1.26 (−6.40 to 3.88)	.627		
Current use of biologic agents	−1.05 (−4.43 to 2.34)	.541		
Number of biologicals	0.18 (−1.08 to 1.44)	.778		
Prior intestinal resection	2.54 (−1.60 to 6.67)	.227		

Abbreviations: BMI, body mass index; CI, confidence interval; CIS, Checklist Individual Strength; IBD, inflammatory bowel disease, IPAQ, International Physical Activity Questionnaire; VAS, visual analog scale.

* Significant *P *< .05.

a Low and medium educational level, including primary education, secondary education, and intermediate vocational education.

b High educational level, including higher vocational education and university.

c Nonemployed, including studying, retired, sick leave, partially or fully unfit to work, or not working for other reasons.

d Employed, including working full-time and working part-time

e Available in n* = *92 in the IBD group, missing data due to responses of “I don’t know.”

f Available in n* = *96 in the IBD group, missing data due to responses of “I don’t know.”

g Available in n* = *102 in the IBD group, missing data due to responses of “I don’t know.”

h Available in n* = *101 in the IBD group, missing data due to responses of “I don’t know.”

i Available in n* = *98 in the IBD group, missing data due to responses of “I don’t know.”

j Clinical disease activity according to the HBI for CD and the SCCAI for UC; active disease was defined as HBI ≥5 or SCCAI ≥3.

k Available in n* = *98 in the IBD group.

l Available in n* = *56 in the IBD group within 4 months around inclusion.

**Table 3.** Univariable and multivariable linear regression analysis of the associations of patient, disease, and treatment characteristics with outcomes from the steep ramp test, sit-to-stand test, and hand-held dynamometry hamstring strength in patients with IBD.

	Cardiorespiratory fitness and muscular strength				Muscular strength							
	Steep ramp test				60-s sit-to-stand test				Hand-held dynamometry hamstring strength			
	Univariable		Multivariable		Univariable		Multivariable		Univariable		Multivariable	
	*B* (95% CI)	*P*	*B* (95% CI)	*P*	*B* (95% CI)	*P*	*B* (95% CI)	*P*	*B* (95% CI)	*P*	*B* (95% CI)	*P*
**Patient characteristics**												
Age at inclusion	−0.04 (−0.05 to −0.03)	<.001^*^	−0.03 (−0.04 to −0.03)	<.001^*^	−0.38 (−0.50 to −0.24)	<.001^*^	−0.32 (−0.43 to −0.20)	<.001^*^	−0.02 (−0.03 to −0.01)	<.001^*^	−0.02 (−0.02 to −0.01)	<.001^*^
Male	0.83 (0.47 to 1.20)	<.001^*^	0.65 (0.44 to 0.86)	<.001^*^	5.84 (1.31 to 10.37)	.012^*^	4.53 (1.03 to 8.04)	.012^*^	0.49 (0.26 to 0.73)	<.001^*^	0.47 (0.28 to 0.66)	<.001^*^
Charlson comorbidity index	−0.51 (−0.67 to −0.34)	<.001^*^			−5.00 (−7.05 to −2.95)	<.001^*^			−0.25 (−0.36 to −0.14)	<.001^*^		
BMI	−0.15 (−0.19 to −0.11)	<.001^*^	−0.11 (−0.13 to −0.08)	<.001^*^	−1.06 (−1.59 to −0.53)	<.001^*^	−0.66 (−1.10 to −0.23)	0.003^*^	−0.08 (−0.10 to −0.05)	<.001^*^	−0.06 (−0.08 to −0.03)	<.001^*^
Malnutrition	0.12 (−0.74 to 0.98)	.783			2.91 (−7.14 to 12,96)	.567			0.07 (−0.48 to 0.62)	.803		
Smoking status												
Nonsmoker	REF	REF			REF	REF			REF	REF		
Current smoker	−0.19 (−0.82 to 0.43)	.541			0.65 (−6.69 to 7.99)	.861			−0.05 (−0.35)	.812		
Educational level												
Low/medium^*^	REF	REF			REF	REF			REF	REF		
High^a^	0.27 (−0.13 to 0.68)	.183			1.28 (−3.49 to 6.04)	.569			−0.01 (−0.27 to 0.25)	.914		
Employment status												
Nonemployed^b^	REF	REF			REF	REF			REF	REF		
Employed^c^	0.54 (0.15 to 0.93)	.008^*^			4.94 (0.29 to 9.59)	.038^*^			0.23 (−0.03 to 0.48)	.080		
CIS total score	−0.01 (−0.02 to 0.00)	.014^*^			−0.12 (−0.21 to −0.03)	.012^*^			0.00 (−0.01 to 0.00)	.207		
CIS subjective fatigue	−0.03 (−0.04 to −0.01)	<.001^*^	−0.02 (−0.03 to −0.01)	<.001^*^	−0.24 (−0.41 to −0.06)	.009^*^			−0.01 (−0.02 to 0.00)	.064		
CIS concentration	−0.01 (−0.03 to 0.02)	.547			−0.16 (−0.45 to 0.14)	.291			0.00 (−0.02 to 0.02)	.955		
CIS motivation	−0.04 (−0.08 to 0.00)	.042^*^			−0.60 (−1.03 to −0.16)	.008^*^	−0.53 (−0.88 to −0.18)	.003^*^	−0.02 (−0.04 to 0.01)	.159		
CIS activity	−0.02 (−0.07 to 0.03)	.345			−0.48 (−1.04 to 0.09)	.096			0.00 (−0.04 to 0.03)	.816		
IPAQ total^d^	0.00 (0.00 to 0.00)	.624			0.00 (0.00 to 0.00)	.137			0.00 (0.00 to 0.00)	.227		
IPAQ walking^e^	0.00 (0.00 to 0.00)	.847			0.00 (0.00 to 0.00)	.204			0.00 (0.00 to 0.00)	.287		
IPAQ moderate intensity^f^	0.00 (0.00 to 0.00)	.963			0.00 (0.00 to 0.00)	.208			0.00 (0.00 to 0.00)	.818		
IPAQ vigorous intensity^g^	0.00 (0.00 to 0.00)	.103			0.00 (0.00 to 0.00)	.148			0.00 (0.00 to 0.00)	.098		
IPAQ sitting^h^	0.01 (0.00 to 0.00)	.467			−0.01 (−0.02 to 0.01)	.283			0.00 (0.00 to 0.00)	.330		
**Disease characteristics**												
Disease entity												
CD	REF	REF			REF	REF			REF	REF		
UC	0.17 (−0.23 to 0.57)	.404			0.48 (−4.20 to 5.15)	.840			0.01 (−0.25 to 0.26)	.968		
Disease duration	−0.03 (−0.04 to −0.01)	.004^*^			−0.22 (−0.42 to −0.01)	.040^*^			−0.01 (−0.02 to 0.00)	.052		
Clinical active disease^i^	−0.35 (−0.81 to 0.12)	.139			−1.56 (−7.04 to 3.91)	.573			−0.03 (−0.33 to 0.27)	.849		
Fecal calprotectin^j^	0.00 (0.00 to 0.00)	.268			0.00 (0.00 to 0.01)	.359			0.00 (0.00 to 0.00)	.475		
IBD-Control-8 score^k^	0.03 (−0.05 to 0.10)	.497			0.55 (−0.26 to 1.36)	.181			0.00 to (−0.04 to 0.05)	.916		
IBD-Control-VAS score^l^	0.01 (−0.01 to 0.02)	.414			0.06 (−0.09 to 0.21)	.414			0.00 (0.00 to 0.01)	.353		
Arthralgia during disease course	−0.64 (−1.06 to 0.22)	.003^*^			−5.32 (−10.34 to −0.31)	.038^*^			−0.37 (−0.64 to −0.10)	.008^*^		
Arthritis during disease course	−0.20 (−0.73 to 0.33)	.448			−8.02 (−14.02 to −2.02)	.009^*^	−8.06 (−12.76 to −3.36)	<.001^*^	−0.26 (−0.59 to 0.07)	.122		
**Treatment characteristics**												
No current medication use	−0.36 (−0.87 to 0.16)	.173			−4.06 (−10.08 to 1.95)	.183			−0.02 (−0.35 to 0.31)	.896		
Current use of mesalazine only	0.07 (−0.46 to 0.60)	.781			−1.18 (−7.38 to 5.01)	.706			−0.09 (−0.42 to 0.25)	.614		
Current use of corticosteroids	0.06 (−0.60 to 0.73)	.849			0.99 (−6.82 to 8.80)	.802			0.03 (−0.39 to 0.46)	.880		
Current use of immunomodulator	0.13 (−0.47 to 0.74)	.669			3.28 (−3.79 to 10.43)	.360			0.03 (−0.36 to 0.41)	.888		
Current use of biologic agents	0.03 (−0.37 to 0.43)	.876			−0.63 (−5.31 to 4.05)	.790			−0.09 (−0.34 to 0.17)	.504		
Number of biologicals	−0.07 (−0.22 to 0.08)	.362			−1.62 (−3.34 to 0.09)	.064	−1.54 (−2.86 to −0.21)	.023^*^	−0.06 (−0.15 to 0.04)	.234		
Prior intestinal resection	−0.42 (−0.90 to 0.07)	.090			−3.45 (−9.15 to 2.25)	.233			−0.08 (−0.39 to 0.24)	.632		

Abbreviations: BMI, body mass index; CD, Crohn’s disease; CI, confidence interval; CIS, Checklist Individual Strength; HBI, Harvey-Bradshaw index; IBD, inflammatory bowel disease, IPAQ, International Physical Activity Questionnaire; SCCAI, Simple Clinical Colitis Activity Index; UC, ulcerative colitis; VAS, visual analog scale.

* Significant *P *< .05.

a Low and medium educational level, including primary education, secondary education, and intermediate vocational education.

b High educational level, including higher vocational education and university.

c Nonemployed, including studying, retired, sick leave, partially or fully unfit to work, or not working for other reasons.

d Employed, including working full-time and working part-time

e Available in n* = *92 in the IBD group, missing data due to responses of “I don’t know.”

f Available in n* = *96 in the IBD group, missing data due to responses of “I don’t know.”

g Available in n* = *102 in the IBD group, missing data due to responses of “I don’t know.”

h Available in n* = *101 in the IBD group, missing data due to responses of “I don’t know.”

i Available in n* = *98 in the IBD group, missing data due to responses of “I don’t know.”

j Clinical disease activity according to the HBI for CD and the SCCAI for UC; active disease was defined as HBI ≥5 or SCCAI ≥3.

k Available in n* = *98 in the IBD group.

l Available in n* = *56 in the IBD group within 4 months around inclusion.

**Table 4.** Univariable and multivariable linear regression analyses of the associations of patient, disease, and treatment characteristics with quadriceps and hamstring muscular endurance indexes in patients with IBD.

	Quadriceps peak torque fatigue index				Hamstring peak torque fatigue index				Quadriceps work fatigue index				Hamstring work fatigue index			
	Univariable		Multivariable		Univariable		Multivariable		Univariable		Multivariable		Univariable		Multivariable	
	*B* (95% CI)	*P*	*B* (95% CI)	*P*	*B* (95% CI)	*P*	*B* (95% CI)	*P*	*B* (95% CI)	*P*	*B* (95% CI)	*p*	*B* (95% CI)	*P*	*B* (95% CI)	*P*
**Patient characteristics**																
Age at inclusion	−0.18 (−0.31 to −0.06)	.005^*^			−0.23 (−0.37 to −0.09)	.001^*^			−0.31 (−0.50 to −0.11)	.003^*^	−0.28 (−0.48 to −0.07)	.008^*^	−0.25 (−0.41 to −0.09)	.002^*^		
Male	−1.05 (−5.14 to 3.03)	.610			−0.39 (−4.92 to 4.15)	.866			−4.73 (−11.14 to 1.67)	.146			−3.48 (−8.65 to 1.69)	.185		
Charlson comorbidity index	−2.41 (−4.34 to −0.48)	.015^*^			−3.75 (−5.83 to −1.67)	<.001^*^	−3.32 (−5.35 to −1.28)	0.002^*^	−3.77 (−6.83 to −0.71)	.016^*^			−4.14 (−6.55 to −1.74)	<.001^*^	−3.68 (−6.05 to −1.31)	.003^*^
BMI	0.36 (−0.14 to 0.85)	.153			0.07 (−0.48 to 0.63)	.791			0.34 (−0.45 to 1.12)	.396			0.06 (−0.58 to 0.69)	.859		
Malnutrition	0.22 (−8.59 to 9.02)	.961			2.04 (−7.18 to 11.79)	.680			1.70 (−12.23 to 15.64)	.809			4.81 (−6.39 to 16.01)	.396		
Smoking status																
Nonsmoker	REF	REF			REF	REF			REF	REF			REF	REF		
Current smoker	4.56 (−1.80 to 10.92)	.158			3.15 (−3.95 to 10.25)	.381			8.12 (−1.93 to 18.16)	.112			0.20 (−8.00 to 8.40)	.961		
Educational level																
Low/medium^*^	REF	REF			REF	REF			REF				REF	REF		
High^a^	−3.50 (−7.62 to 0.61)	.095			1.59 (−3.02 to 6.21)	.495			−5.66 (−12.18 to 0.85)	.088			3.38 (−1.91 to 8.66)	.208		
Employment status																
Nonemployed^b^	REF	REF			REF	REF			REF	REF	REF		REF	REF		
Employed^c^	−2.75 (−6.87 to 1.37)	.189			0.99 (−3.62 to 5.59)	.672			−7.08 (−13.51 to −0.65)	.031^*^	−6.78 (−13.12 to −0.44)	.036^*^	−2.72 (−8.00 to 2.56)	.310		
CIS total score	0.08 (0.00 to 0.16)	.057			0.14 (0.06 to 0.23)	.002^*^			0.18 (0.05 to 0.30)	.006^*^			0.14 (0.04 to 0.25)	.005^*^		
CIS subjective fatigue	0.19 (0.03 to 0.34)	.019^*^			0.27 (0.10 to 0.44)	.002^*^	0.23 (0.07 to 0.39)	.006^*^	0.39 (0.15 to 0.63)	.002^*^	0.25 (0.00 to 0.49)	.047^*^	0.29 (0.09 to 0.48)	.004^*^	0.24 (0.06 to 0.43)	.012^*^
CIS concentration	0.20 (−0.05 to 0.46)	.116			0.34 (0.06 to 0.61)	.018^*^			0.48 (0.08 to 0.87)	.019^*^			0.39 (0.08 to 0.71)	.016^*^		
CIS motivation	0.19 (−0.20 to 0.58)	.333			0.48 (0.05 to 0.90)	.029^*^			0.34 (−0.28 to 0.96)	.276			0.35 (−0.14 to 0.85)	.162		
CIS activity	0.00 (−0.50 to 0.50)	.993			0.57 (0.03 to 1.11)	.038^*^			0.38 (−0.40 to 1.17)	.336			0.45 (−0.18 to 1.09)	.156		
IPAQ total^d^	0.00 (0.00 to 0.00)	.680			0.00 (0.00 to 0.00)	.437			0.00 (0.00 to 0.00)	.298			0.00 (0.00 to 0.00)	.994		
IPAQ walking^e^	0.00 (0.00 to 0.00)	.047^*^			0.00 (0.00 to 0.00)	.123			0.00 (0.00 to 0.00)	.075			0.00 (0.00 to 0.00)	.259		
IPAQ moderate intensity^f^	0.00 (0.00 to 0.00)	.747			0.00 (0.00 to 0.00)	.848			0.00 (0.00 to 0.00)	.875			0.00 (0.00 to 0.00)	.508		
IPAQ vigorous intensity^g^	0.00 (0.00 to 0.00)	.998			0.00 (−0.01 to 0.01)	.824			0.00 (−0.01 to 0.01)	.697			0.00 (−0.01 to 0.01)	.796		
IPAQ sitting^h^	0.00 (−0.01 to 0.01)	.507			0.01 (−0.01 to 0.02)	.294			0.00 (−0.02 to 0.02)	.988			0.01 (−0.01 to 0.02)	.235		
**Disease characteristics**																
Disease entity																
CD	REF	REF	REF		REF				REF	REF			REF	REF		
UC	−6.89 (−10.75 to −3.03)	<.001^*^	−5.82 (−10.02 to −1.62)	.007^*^	−2.36 (−6.87 to 2.15)	0.302			−9.17 (−15.39 to −2.94)	.004^*^			−3.16 (−8.35 to 2.03)	.230		
Disease duration	−0.20 (−0.38 to −0.02)	.034^*^	−0.17 (−0.36 to 0.02)	.071	−0.10 (−0.30 to 0.10)	.328			−0.30 (−0.59 to −0.02)	.037^*^			−0.11 (−0.34 to 0.13)	.363		
Clinical active disease^i^	3.60 (−1.15 to 8.34)	.136			3.68 (−1.59 to 8.95)	.169			7.46 (0.01 to 14.92)	.050			5.75 (−0.28 to 11.77)	.061		
Fecal calprotectin^j^	0.01 (0.00 to 0.01)	.043^*^	0.00 (0.00 to 0.01)	0.055	0.00 (0.00 to 0.01)	.233			0.00 (0.00 to 0.01)	.310			0.00 (0.00 to 0.01)	.322		
IBD-Control-8 score^k^	−0.77 (−1.54 to −0.01)	.049^*^			−0.44 (−1.31 to 0.42)	.308			−1.64 (−2.91 to −0.39)	.011^*^			−0.79 (−1.86 to 0.28)	.144		
IBD-Control-VAS score^l^	−0.09 (−0.23 to 0.05)	.219			−0.03 (−0.19 to 0.13)	.692			−0.21 (−0.45 to 0.02)	.069			−0.08 (−0.27 to 0.11)	.411		
Arthralgia during disease course	0.24 (−4.24 to 4.72)	.917			0.20 (−4.77 to 5.17)	.937			−2.83 (−9.91 to 4.24)	.429			0.57 (−5.15 to 6.29)	.845		
Arthritis during disease course	−2.58 (−7.98 to 2.82)	.346			−0.12 (−6.13 to 5.90)	.969			−3.10 (−11.66 to 5.47)	.475			1.98 (−4.93 to 8.89)	.572		
**Treatment characteristics**																
No current medication use	−1.76 (−7.06 to 3.54)	.511			4.95 (−0.85 to 10.76)	.094			−1.78 (−10.18 to 6.62)	.676			3.86 (−2.87 to 10.60)	.258		
Current use of mesalazine only	−7.84 (−13.04 to −2.63)	.004^*^	−6.66 (−12.26 to −1.07)	0.020^*^	−6.88 (−12.74 to −1.02)	.022^*^			−12.97 (−21.18 to −4.77)	.002^*^	−11.70 (−20.00 to −3.41)	.006^*^	−5.56 (−12.39 to 1.28)	.110		
Current use of corticosteroids	2.24 (−4.58 to 9.06)	.516			−2.38 (−9.95 to 5.18)	.533			5.99 (−4.77 to 16.75)	.272			−0.05 (−8.77 to 8.68)	.991		
Current use of immunomodulator	1.83 (−4.36 to 8.02)	.559			−2.02 (−8.89 to 4.85)	.561			3.40 (−6.41 to 13.20)	.493			−2.63 (−10.53 to 5.28)	.512		
Current use of biologic agents	4.10 (0.09 to 812)	.045^*^			1.85 (−2.68 to 6.37)	.420			6.56 (0.21 to 12.91)	.043^*^			2.42 (−2.79 to 7.62)	.359		
Number of biologicals	0.87 (−0.65 to 2.38)	.259			0.66 (−1.03 to 2.34)	.442			1.24 (−1.16 to 3.64)	.308			0.13 (−1.82 to 2.07)	.899		
Prior intestinal resection	2.33 (−2.67 to 7.33)	.358			−0.90 (−6.47 to 4.67)	.749			2.04 (−5.90 to 9.98)	.611			−1.68 (−8.08 to 4.73)	.605		

Abbreviations: BMI, body mass index; CD, Crohn’s disease; CI, confidence interval; CIS, Checklist Individual Strength; HBI, Harvey-Bradshaw index; IBD, inflammatory bowel disease, IPAQ, International Physical Activity Questionnaire; SCCAI, Simple Clinical Colitis Activity Index; UC, ulcerative colitis; VAS, visual analog scale.

* Significant P < .05.

a Low and medium educational level, including primary education, secondary education, and intermediate vocational education.

b High educational level, including higher vocational education and university.

c Nonemployed, including studying, retired, sick leave, partially or fully unfit to work, or not working for other reasons.

d Employed, including working full-time and working part-time

e Available in n* = *92 in the IBD group, missing data due to responses of “I don’t know.”

f Available in n* = *96 in the IBD group, missing data due to responses of “I don’t know.”

g Available in n* = *102 in the IBD group, missing data due to responses of “I don’t know.”

h Available in n* = *101 in the IBD group, missing data due to responses of “I don’t know.”

i Available in n* = *98 in the IBD group, missing data due to responses of “I don’t know.”

j Clinical disease activity according to the HBI for CD and the SCCAI for UC; active disease was defined as HBI ≥5 or SCCAI ≥3.

k Available in n* = *98 in the IBD group.

l Available in n* = *56 in the IBD group within 4 months around inclusion. In case of *P *< .10 in the univariable analysis, a separate multivariable model was conducted to prevent substantial data loss for the remaining variables.

**Table 5.** Univariable and multivariable linear regression analyses of the associations of patient, disease, and treatment characteristics with distance reached with the sit-and-reach test in patients with IBD.

	Univariable		Multivariable	
	*B* (95% CI)	*P*	*B* (95% CI)	*P*
**Patient characteristics**				
Age at inclusion	−0.09 (−0.22 to 0.04)	.169		
Male	−8.34 (−12.02 to −4.67)	<.001^*^	−8.46 (−12.06 to −4.85)	<.001^*^
Charlson comorbidity index	−0.65 (−2.60 to 1.30)	.511		
BMI	−0.46 (−0.94 to 0.03)	.063	−0.49 (−0.92 to −0.05)	.031^*^
Malnutrition	3.40 (−5.23 to 12.03)	.437		
Smoking status				
Nonsmoker	REF	REF		
Current smoker	3.90 (−2.37 to 10.17)	.220		
Educational level				
Low/medium^a^	REF	REF		
High^b^	−1.64 (−5.73 to 2.45)	.427		
Employment status				
Nonemployed^c^	−1.60 (−5.67 to 2.47)	REF		
Employed^d^	−0.08	.438		
CIS total score	0.03 (−0.06 to 0.11)	.539		
CIS subjective fatigue	0.06 (−0.10 to 0.21)	.471		
CIS concentration	0.14 (−0.11 to 0.39)	.264		
CIS motivation	−0.16 (−0.55 to 0.23)	.410		
CIS activity	0.09 (−0.40 to 0.58)	.723		
IPAQ total^e^	0.00 (0.00 to 0.00)	.808		
IPAQ walking^f^	0.00 (0.00 to 0.00)	.581		
IPAQ moderate intensity^g^	0.00 (0.00 to 0.00)	.781		
IPAQ vigorous intensity^h^	0.00 (0.00 to 0.00)	.884		
IPAQ sitting^i^	−0.01 (−0.01 to 0.01)	.354		
**Disease characteristics**				
Disease entity				
CD	REF	REF		
UC	0.88 (−3.15 to 4.89)	.667		
Disease duration	−0.16 (−0.34 to 0.02)	.074		
Clinical active disease^j^	3.20 (−1.48 to 7.88)	.178		
Fecal calprotectin^k^	0.00 (0.00 to 0.01)	.814		
IBD-Control-8 score^l^	−0.62 (−1.36 to 0.11)	.095		
IBD-Control-VAS score^l^	−0.12 (−0.25 to 0.02)	.083		
Arthralgia during disease course	0.70 (−3.71 to 5.10)	.755		
Arthritis during disease course	0.45 (−4.88 to 5.78)	.868		
**Treatment characteristics**				
No current medication use	0.05 (−5.17 to 5.27)	.986		
Current use of mesalazine only	−1.97 (−7.28 to 3.35)	.465		
Current use of corticosteroids	4.29 (−2.37 to 10.96)	.204		
Current use of immunomodulator	3.64 (−2.42 to 9.70)	.236		
Current use of biologic agents	−1.33 (−5.35 to 2.68)	.512		
Number of biologicals	−0.81 (−2.30 to 0.68)	.283		
Prior intestinal resection	−1.91 (−6.83 to 3.02)	.444		

Abbreviations: BMI, body mass index; CD, Crohn’s disease; CI, confidence interval; CIS, Checklist Individual Strength; HBI, Harvey-Bradshaw index; IBD, inflammatory bowel disease, IPAQ, International Physical Activity Questionnaire; SCCAI, Simple Clinical Colitis Activity Index; UC, ulcerative colitis; VAS, visual analog scale.

* Significant *P *< .05.

a Low and medium educational level, including primary education, secondary education, and intermediate vocational education.

b High educational level, including higher vocational education and university.

c Nonemployed, including studying, retired, sick leave, partially or fully unfit to work, or not working for other reasons.

d Employed, including working full-time and working part-time

e Available in n* = *92 in the IBD group, missing data due to responses of “I don’t know.”

f Available in n* = *96 in the IBD group, missing data due to responses of “I don’t know.”

g Available in n* = *102 in the IBD group, missing data due to responses of “I don’t know.”

h Available in n* = *101 in the IBD group, missing data due to responses of “I don’t know.”

i Available in n* = *98 in the IBD group, missing data due to responses of “I don’t know.”

j Clinical disease activity according to the HBI for CD and the SCCAI for UC; active disease was defined as HBI ≥5 or SCCAI ≥3.

k Available in n* = *98 in the IBD group.

l Available in n* = *56 in the IBD group within 4 months around inclusion. In case of *P *< .10 in the univariable analysis, a separate multivariable model was conducted to prevent substantial data loss for the remaining variables.

### Patients’ perspectives on physical fitness

**Figure 2.**
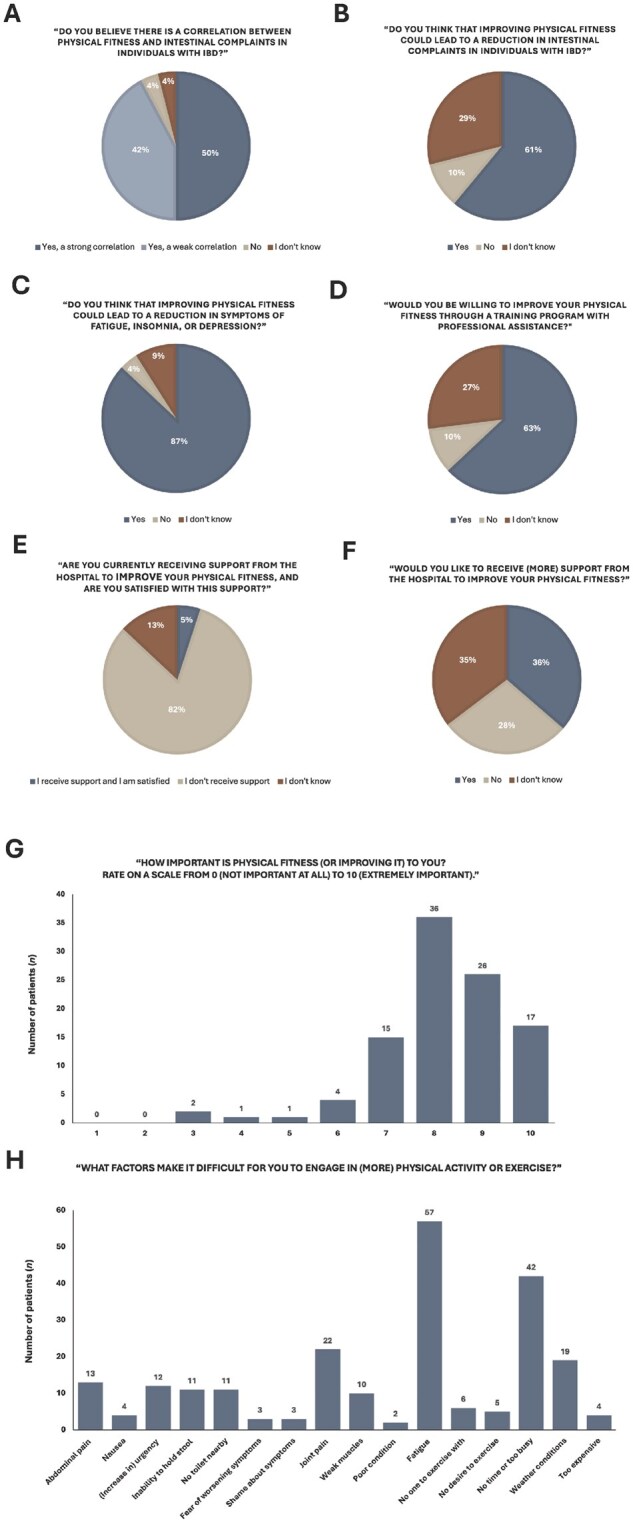
Patients’ perspectives on physical fitness.

Figure 2 illustrates responses to questions on patients’ perspectives regarding physical fitness. Nearly all patients (92%) believed in a correlation between physical fitness and intestinal symptoms in IBD, with 61% believing that improved physical fitness could alleviate these symptoms and 87% expecting it to reduce fatigue, insomnia, or depression. The majority (63%) expressed willingness to improve their fitness with professional support, though only 5% currently received support from the hospital, and 36% indicated they would like to receive (more) support to improve their physical fitness. Over 77% of patients rated the importance of (improving) their physical fitness as 8 or higher on a scale of 0 to 10. The most commonly described barrier to physical activity or physical exercise training was fatigue (n* = *57), followed by lack of time (n* = *42), joint pain (n* = *22), and weather conditions (n* = *19).

## Discussion

This is the first study to comprehensively assess and compare the 5 components of health-related physical fitness in patients with IBD compared with a matched healthy control group. Our findings demonstrate that patients with IBD have higher body fat percentages and reduced cardiorespiratory fitness and muscular strength compared with healthy control subjects, while no significant differences were observed in muscular endurance or flexibility. Additionally, various independent disease- and treatment-specific factors associated with health-related physical fitness in IBD were identified, suggesting subgroups within the IBD population who may be at particularly high risk of physical fitness deficits, pointing to the potential value of personalized physical exercise training interventions. Finally, most patients consider physical fitness important and potentially beneficial for their symptoms, with the majority expressing interest in professional support to improve their physical fitness.

The higher body fat percentage observed in patients with IBD in our study is consistent with prior research indicating a rising prevalence of overweight and obesity within the IBD population.^28^ This contradicts the misconception that the majority of patients with IBD are underweight. The exact mechanism driving these body composition alterations in IBD are not yet fully understood. However, contributing factors likely include unhealthy lifestyle habits such as low physical activity levels and high-calorie diets, glucocorticoid therapy, imbalances in gut microbiota, and altered metabolic signaling in the gut mediated by hormones.^29^^,^^30^ Adiposity has been shown to impact disease activity and severity, especially in patients with CD, where specific fat measurements, such as visceral adipose tissue, seem to be stronger predictors than traditional BMI.^28^^,^^31^ Elevated levels of visceral adipose tissue levels were previously associated with increased surgery rates, risk of penetrating disease, and postoperative morbidity in patients with CD.^32–34^ Furthermore, obesity may also reduce the efficacy of biologics, particularly tumor necrosis factor α agents, by increasing drug clearance and distribution volume.^28^ In our study, BMI was consistently associated with increased body fat percentage and reductions in cardiorespiratory fitness and muscular strength, suggesting its potential as clinical measure impacting components of health-related physical fitness.

Our findings of reduced cardiorespiratory fitness in patients with IBD, measured by steep ramp test performance, build upon previous evidence reporting similar results.^19^^,^^35^^,^^36^ Impaired cardiorespiratory fitness was previously shown in patients with CD in remission and in a cohort of IBD patients awaiting surgery.^35^^,^^36^ The reduction in cardiorespiratory fitness found in the current study could partly be explained by increased levels of circulating proinflammatory cytokines.^37^^,^^38^ Additionally, it could reflect a more sedentary lifestyle in patients with IBD. Although we observed no significant difference in total physical activity levels between groups, patients engaged significantly less in vigorous-intensity activity, which is crucial for maintaining and enhancing cardiorespiratory fitness.^9^ This suggests that while overall activity levels may be comparable, individuals with IBD are more likely to opt for lower-intensity forms of physical activity. Additionally, higher fatigue levels in the IBD group likely further reduce engagement in (vigorous) physical activity, as fatigue was commonly reported as a barrier to increasing physical activity or exercise in our study. The inverse association between cardiorespiratory fitness and fatigue is consistent with findings by Vogelaar et al,^5^ and further highlights fatigue as a key factor limiting cardiorespiratory fitness in patients with IBD.

The reduced performance on the steep ramp test in patients with IBD also reflects decreased muscular strength, which was further supported by lower scores at the 60-second sit-to-stand test and hand-held dynamometry for hamstring strength. Previous studies on muscular strength in IBD have yielded inconsistent results, likely due to differences in testing methods, patient characteristics, and adjustments for body mass, complicating direct comparisons across studies.^19^ Emerging research suggests a gut-muscle axis, in which intestinal inflammation, gut dysbiosis, and malnutrition may contribute to muscle loss in IBD.^39^^,^^40^ Further, chronic intestinal inflammation and the release of proinflammatory cytokines (eg, tumor necrosis factor α, interleukine-6), can impair muscle function by activating protein-degrading pathways, such as the nuclear factor κB and JAK/STAT pathways. Additionally, gut microbiota imbalances, frequently observed in IBD, could compromise the intestinal barrier among others, further promoting inflammation and muscle degradation. Furthermore, chronic inflammation or intolerances in IBD may reduce appetite and food intake, leading to deficiencies in micronutrients or insufficient protein intake, both essential for muscle health. Also, the use of corticosteroids in the disease course, which are known for their myopathic effects, could contribute to impairments in muscular strength among these patients.^3^^,^^41^ In the present study, fatigue, arthritis during the disease course, and a higher number of different biologicals used was inversely associated with muscular strength. While fatigue and (previous) arthritis are likely to restrict physical activity and exercise levels, which are crucial for maintaining muscular strength, a higher number of biologics may reflect a more aggressive, therapy-resistant disease, in which persistent inflammation could exacerbate muscle breakdown.^42^

The absence of significant differences in muscular endurance and flexibility suggests that these physical fitness components may be relatively preserved in patients with IBD. To our knowledge, no prior studies have assessed flexibility in patients with IBD, making these findings novel. Regarding muscular endurance, multivariable linear regression and subgroup analyses indicated a more pronounced reduction in quadriceps muscular endurance specifically among patients with CD compared with those with UC. Only 2 previous studies have examined muscular endurance in patients with CD compared with healthy control subjects using isometric or isokinetic dynamometry, yielding conflicting results.^43^^,^^44^ Salacinski et al^43^ reported better endurance of the rectus femoris muscle and comparable endurance of the vastus lateralis muscle among CD patients in remission with prior small bowel resection and musculoskeletal issues, whereas van Langenberg et al^44^ showed reduced knee extensor endurance. The alignment of our findings with the latter may stem from the use of similar testing protocols, as muscular endurance seems to be highly task specific.^45^ Consistent with van Langenberg et al, we also found an association between reduced muscular endurance and fatigue. Additionally, our study identified associations with unemployment, a higher Charlson comorbidity index, and the use of medications beyond mesalazine, all likely reflecting greater (comorbid) disease burden that may limit physical activity and contribute to muscle deconditioning.

Our findings carry important clinical implications. The identified increases in body fat and impairments in cardiorespiratory fitness and muscular strength underscore the impact of IBD on health-related physical fitness. Nonetheless, the differences in health-related physical fitness identified between the overall IBD population and healthy control subjects were relatively small. This suggests that it is important to properly preselect patients with significant deficits in health-related physical fitness components who may therefore benefit most from targeted physical exercise interventions. Future research should focus on establishing age- and sex-specific reference values in large populations to determine cutoff points for the physical fitness tests employed in this study. Such preselection could be further informed by disease- and treatment-related factors associated with fitness impairment, as identified in this study. To better understand whether these physical fitness impairments are specific to IBD or reflect more general patterns seen across chronic conditions, future studies should also consider including comparator groups with other (chronic) diseases. For example, individuals with irritable bowel syndrome, which shares gastrointestinal symptoms but lacks chronic inflammation, could provide valuable insight into the role of inflammation in the development of fitness impairments. Similarly, comparisons with patients with rheumatoid arthritis, which is also a chronic inflammatory disease associated with functional limitations, may help distinguish between shared and disease-specific mechanisms underlying reduced physical fitness. Such comparisons could also inform whether physical exercise interventions developed for other patient populations might be effectively adapted for use in IBD care. Furthermore, longitudinal studies are warranted to determine whether tailored physical exercise training interventions can effectively improve specific components of health-related fitness in unfit patients with IBD. In addition, such studies should evaluate whether improvements in these physical fitness components correlate with better disease control, improved patient-reported outcomes, and reduced postoperative morbidity. To effectively implement such interventions, understanding patients’ perspectives on physical fitness is essential, including their specific needs and challenges. Our study revealed that most patients consider physical fitness to be important and believe it could benefit their intestinal symptoms, but also symptoms of fatigue, insomnia, and depression, with many expressing interests in professional support to improve their physical fitness. When developing exercise-based interventions, it is crucial to address commonly reported barriers, such as fatigue, lack of time, and joint pain. Engaging patients in the design and implementation of such interventions can improve effectiveness and promote adherence.

This study is the first to comprehensively assess all 5 components of health-related physical fitness in patients with IBD compared with healthy control subjects, offering an in-depth understanding of how IBD affects overall physical fitness. A large sample of patients and healthy control subjects was included and validated measurement methods were used to assess the components of physical fitness, which strengthens the robustness of our findings.^20^ Moreover, we assessed nutritional status as a potential determinant of physical fitness using the validated Global Leadership Initiative on Malnutrition criteria. However, our study also has some limitations. First, the lower statistical power for CD and UC subgroups analyses may have prevented the detection of clinically meaningful differences and associations for some health-related physical fitness measures. Second, we did not correct for multiple testing in this study to minimize the risk of overlooking clinically relevant associations. Third, the cross-sectional design of our study allowed us to identify associations but does not establish causation between IBD and reduced physical fitness. Fourth, as our populations consisted of volunteer participants, the findings may overestimate physical fitness and physical activity levels, as volunteers for the current study may be more interested in physical fitness and may be more physically active than the broader IBD and general population. This volunteer bias may have been more pronounced in the control group. Although all patients were encouraged to invite a control participant from their personal network, additional recruitment via public advertisements was required. This may have contributed to the demographic differences observed between patients and healthy control subjects, suggesting a higher socioeconomic status in healthy control subjects (eg, lower former smoking rates, higher educational levels). While we adjusted for these variables in our analyses and the observed differences in health-related physical fitness outcomes between groups remained robust, employing a more refined control matching strategy might have further reduced bias and enhanced group comparability. Future studies could consider matched-pair designs or population-based sampling approaches to further strengthen representativeness and minimize potential confounding. Sixth, we did not collect data on participants’ ethnicity, which may have limited our ability to explore potential ethnic differences in physical fitness. Finally, we relied on self-reported physical activity, which is more susceptible to bias compared with objective measures such as accelerometers.

## Conclusion

In this systematic evaluation of health-related physical fitness components, specific impairments among patients with IBD were observed, including higher body fat and reduced cardiorespiratory fitness and muscular strength compared with healthy control subjects. Particularly, those with a higher age, with female sex, with higher BMI, experiencing fatigue or arthritis during the disease course, and who have been treated with multiple biologic agents are at risk for impairments in health-related physical fitness and might benefit from targeted physical exercise training interventions aimed at enhancing these impairments. Future longitudinal studies are warranted to evaluate the effectiveness of tailored training interventions on improving physical fitness components and to determine whether these improvements translate into better disease outcomes and quality of life.

## Supplementary Material

izaf169_Supplementary_Data

## Data Availability

The datasets generated and analyzed during the present study are available from the corresponding author upon reasonable request.

## References

[1] Burisch J , Jess T, Martinato M, Lakatos PL; ECCO -EpiCom. The burden of inflammatory bowel disease in Europe. J Crohns Colitis. 2013;7:322-337. 23395397 10.1016/j.crohns.2013.01.010

[2] Romberg-Camps MJL , Bol Y, Dagnelie PC, et al Fatigue and health-related quality of life in inflammatory bowel disease: results from a population-based study in the Netherlands: the ibd-South Limburg cohort. Inflamm Bowel Dis. 2010;16:2137-2147. 20848468 10.1002/ibd.21285

[3] Schakman O , Gilson H, Thissen JP. Mechanisms of glucocorticoid-induced myopathy. J Endocrinol. 2008;197:1-10. 18372227 10.1677/JOE-07-0606

[4] Ünal NG , Oruç N, Tomey O, Ömer Özütemiz A. Malnutrition and sarcopenia are prevalent among inflammatory bowel disease patients with clinical remission. Eur J Gastroenterol Hepatol. 2021;33:1367-1375. 33470696 10.1097/MEG.0000000000002044

[5] Vogelaar L , van den Berg-Emons R, Bussmann H, et al Physical fitness and physical activity in fatigued and nonfatigued inflammatory bowel disease patients. Scand J Gastroenterol. 2015;50:1357-1367. 25966749 10.3109/00365521.2015.1046135

[6] Fagan G , Osborne H, Schultz M. Physical activity in patients with inflammatory bowel disease: a cross-sectional study. Inflamm Intest Dis. 2021;6:61-69. 34124177 10.1159/000511212 PMC8160568

[7] Caspersen CJ , Powell KE, Christenson GM. Physical activity, exercise, and physical fitness: definitions and distinctions for health-­related research. Public Health Rep. 1985;100:126-131. 3920711 PMC1424733

[8] Bouchard C , Shephard RJ. Physical activity, fitness, and health: the model and key concepts. In: C Bouchard, Shephard RJ, T Stephens, eds. Physical Activity, Fitness, and Health: International Proceedings and Consensus Statement. Human Kinetics Publishers; 1994:77-88.

[9] Ross R , Blair SN, Arena R, et al Stroke Council. Importance of assessing cardiorespiratory fitness in clinical practice: a case for fitness as a clinical vital sign: a scientific statement from the american heart association. Circulation. 2016;134:e653-e699. 27881567 10.1161/CIR.0000000000000461

[10] García-Hermoso A , Cavero-Redondo I, Ramírez-Vélez R, et al Muscular strength as a predictor of all-cause mortality in an apparently healthy population: a systematic review and meta-­analysis of data from approximately 2 million men and women. Arch Phys Med Rehabil. 2018;99:2100-2113.e5. 29425700 10.1016/j.apmr.2018.01.008

[11] Cuijpers ACM , Heldens AFJM, Bours MJL, et al Relation between preoperative aerobic fitness estimated by steep ramp test performance and postoperative morbidity in colorectal cancer surgery: prospective observational study. Br J Surg. 2022;109:155-159. 34536001 10.1093/bjs/znab292 PMC10364754

[12] Berkel AEM , Bongers BC, Kotte H, et al Effects of community-­based exercise prehabilitation for patients scheduled for colorectal surgery with high risk for postoperative complications: results of a randomized clinical trial. Ann Surg. 2022;275:e299-e306. 33443905 10.1097/SLA.0000000000004702 PMC8746915

[13] Barberan-Garcia A , Ubré M, Roca J, et al Personalised prehabilitation in high-risk patients undergoing elective major abdominal surgery: a randomized blinded controlled trial. Ann Surg 2018;267:50-56. 28489682 10.1097/SLA.0000000000002293

[14] van Erp LW , Roosenboom B, Komdeur P, et al Improvement of fatigue and quality of life in patients with quiescent inflammatory bowel disease following a personalized exercise program. Dig Dis Sci. 2021;66:597-604. 32239380 10.1007/s10620-020-06222-5

[15] Loudon CP , Corroll V, Butcher J, Rawsthorne P, Bernstein CN. The effects of physical exercise on patients with Crohn’s disease. Am J Gastroenterol. 1999;94:697-703. 10086654 10.1111/j.1572-0241.1999.00939.x

[16] Ng V , Millard W, Lebrun C, Howard J. Low-intensity exercise improves quality of life in patients with Crohn’s disease. Clin J Sport Med. 2007;17:384-388. 17873551 10.1097/JSM.0b013e31802b4fda

[17] Jones K , Kimble R, Baker K, Tew GA. Effects of structured exercise programmes on physiological and psychological outcomes in adults with inflammatory bowel disease (ibd): a systematic review and meta-analysis. PLoS One 2022;17:e0278480. 36454911 10.1371/journal.pone.0278480 PMC9714897

[18] Jones K , Baker K, Speight RA, Thompson NP, Tew GA. ­Randomised clinical trial: combined impact and resistance training in adults with stable Crohn’s disease. Aliment Pharmacol Ther. 2020;52:964-975. 33119156 10.1111/apt.16002

[19] Demers K , Bak MTJ, Bongers BC, et al Scoping review on health-related physical fitness in patients with inflammatory bowel disease: assessment, interventions, and future directions. World J Gastroenterol. 2023;29:5406-5427. 37900583 10.3748/wjg.v29.i38.5406 PMC10600796

[20] Demers K , Bongers BC, van Kuijk SMJ, et al Criterion validity of screening tools and field-based tests for health-related physical fitness in inflammatory bowel disease. Dig Dis Sci. 2024;69:4072-4088. 39425857 10.1007/s10620-024-08682-5 PMC11567995

[21] Vercoulen JAM , Bleijenberg G. De Checklist Individual Strength (cis). Gedragstherapie. 1999;32:131-136.

[22] Craig CL , Marshall AL, Sjöström M, et al International physical activity questionnaire: 12-country reliability and validity. Med Sci Sports Exerc. 2003;35:1381-1395. 12900694 10.1249/01.MSS.0000078924.61453.FB

[23] Cederholm T , Jensen GL, Correia MITD, et al; GLIM Working Group. Glim criteria for the diagnosis of malnutrition—a consensus report from the global clinical nutrition community. Clinical Nutrition. 2019;38:1-9. 30181091 10.1016/j.clnu.2018.08.002

[24] Kurik G , Kelly-Bissue C, Lõhmus A, et al Standardising and simplifying the Global Leadership Initiative on Malnutrition (glim) for its more general application. Clin Nutr ESPEN. 2024;62:120-127. 38901933 10.1016/j.clnesp.2024.05.010

[25] Charlson ME , Pompei P, Ales KL, MacKenzie CR. A new method of classifying prognostic comorbidity in longitudinal studies: development and validation. J Chronic Dis. 1987;40:373-383. 3558716 10.1016/0021-9681(87)90171-8

[26] de Jong MJ , van der Meulen-de Jong AE, Romberg-Camps MJ, et al Telemedicine for management of inflammatory bowel disease (MyIBDcoach): a pragmatic, multicentre, randomised controlled trial. Lancet. 2017;390:959-968. 28716313 10.1016/S0140-6736(17)31327-2

[27] Bodger K , Ormerod C, Shackcloth D, Harrison M, IBD Control Collaborative Development and validation of a rapid, generic measure of disease control from the patient’s perspective: the ibd-­Control questionnaire. Gut. 2014;63:1092-1102. 24107590 10.1136/gutjnl-2013-305600 PMC4078750

[28] Peraza J , Abbott E, Shneyderman M, et al The rising epidemic of obesity in patients with inflammatory bowel disease. Curr Treat Options Gastroenterol. 2024;22:134-144.

[29] Kim JH , Oh CM, Yoo JH. Obesity and novel management of inflammatory bowel disease. World J Gastroenterol. 2023;29:1779-1794. 37032724 10.3748/wjg.v29.i12.1779 PMC10080699

[30] Singh S , Dulai PS, Zarrinpar A, Ramamoorthy S, Sandborn WJ. Obesity in ibd: epidemiology, pathogenesis, disease course and treatment outcomes. Nat Rev Gastroenterol Hepatol. 2017;14:110-121. 27899815 10.1038/nrgastro.2016.181 PMC5550405

[31] Ding NS , Tassone D, Al Bakir I, et al Systematic review: the impact and importance of body composition in inflammatory bowel disease. J Crohns Colitis. 2022;16:1475-1492. 35325076 10.1093/ecco-jcc/jjac041 PMC9455788

[32] Van Der Sloot KWJ , Joshi AD, Bellavance DR, et al Visceral adiposity, genetic susceptibility, and risk of complications among individuals with Crohn’s disease. Inflamm Bowel Dis. 2017;23:82-88. 27893544 10.1097/MIB.0000000000000978 PMC5177457

[33] Connelly TM , Juza RM, Sangster W, et al Volumetric fat ratio and not body mass index is predictive of ileocolectomy outcomes in Crohn’s disease patients. Dig Surg. 2014;31:219-224. 25277149 10.1159/000365359

[34] Stidham RW , Waljee AK, Day NM, et al Body fat composition assessment using analytic morphomics predicts infectious complications after bowel resection in Crohn’s disease. Inflamm Bowel Dis. 2015;21:1306-1313. 25822011 10.1097/MIB.0000000000000360 PMC4437863

[35] Nguyen T , Ploeger HE, Obeid J, et al Reduced fat oxidation rates during submaximal exercise in adolescents with Crohn’s disease. Inflammatory Bowel Dis. 2013;19:2659-2665. 10.1097/01.MIB.0000436958.54663.4f 24105390

[36] Otto JM , O’Doherty AF, Hennis PJ, et al Preoperative exercise capacity in adult inflammatory bowel disease sufferers, determined by cardiopulmonary exercise testing. Int J Colorectal Dis. 2012;27:1485-1491. 22842663 10.1007/s00384-012-1533-4

[37] Robson-Ansley PJ , de Milander L, Collins M, Noakes TD. Acute interleukin-6 administration impairs athletic performance in healthy, trained male runners. Can J Appl Physiol. 2004;29:411-418. 15317982 10.1139/h04-026

[38] Sanchez-Munoz F , Dominguez-Lopez A, Yamamoto-Furusho JK. Role of cytokines in inflammatory bowel disease. World J Gastroenterol. 2008;14:4280-4288. 18666314 10.3748/wjg.14.4280 PMC2731177

[39] Nardone OM , de Sire R, Petito V, et al Inflammatory bowel diseases and sarcopenia: the role of inflammation and gut microbiota in the development of muscle failure. Front Immunol. 2021;12:694217. 34326845 10.3389/fimmu.2021.694217 PMC8313891

[40] de Sire R , Rizzatti G, Ingravalle F, et al Skeletal muscle-gut axis: emerging mechanisms of sarcopenia for intestinal and extra intestinal diseases. Minerva Gastroenterol Dietol. 2018;64:351-362. 30016852 10.23736/S1121-421X.18.02511-4

[41] Mitsui T , Azuma H, Nagasawa M, et al Chronic corticosteroid administration causes mitochondrial dysfunction in skeletal muscle. J Neurol. 2002;249:1004-1009. 12195445 10.1007/s00415-002-0774-5

[42] Dhaliwal A , Quinlan JI, Overthrow K, et al Sarcopenia in inflammatory bowel disease: a narrative overview. Nutrients. 2021;13:656. 33671473 10.3390/nu13020656 PMC7922969

[43] Salacinski AJ , Regueiro MD, Broeder CE, McCrory JL. Decreased neuromuscular function in Crohn’s disease patients is not associated with low serum vitamin D levels. Dig Dis Sci. 2013;58:526-533. 22949179 10.1007/s10620-012-2372-4

[44] van Langenberg DR , Della Gatta P, Warmington SA, et al Objectively measured muscle fatigue in Crohn’s disease: correlation with self-reported fatigue and associated factors for clinical application. J Crohns Colitis. 2014;8:137-146. 23938210 10.1016/j.crohns.2013.07.006

[45] Enoka RM , Stuart DG. Neurobiology of muscle fatigue. J Appl Physiol (1985). 1992;72:1631-1648. 1601767 10.1152/jappl.1992.72.5.1631

