# Supplementary material for: Health-Related Physical Fitness in Patients With Inflammatory Bowel Disease vs Healthy Control Subjects
Source: Inflamm Bowel Dis. 2025 Oct 8;31(12):3337–55. doi: 10.1093/ibd/izaf169 (PMC12688079; doi:10.1093/ibd/izaf169)
Supplement: izaf169_Supplementary_Data [file izaf169_supplementary_data.docx]

**Supplementary materials**

**Supplementary Table 1.** Characteristics of patients with CD (*n* = 55), patients with UC (*n* = 50), and healthy controls (*n* = 102)

| Demographic and clinical characteristics | CD  (*n* = 55) | UC  (*n* = 50) | HC  (*n* = 102) | *p*  (CD *vs* UC) | *p*  (CD *vs* HC,  UC *vs* HC) |
| --- | --- | --- | --- | --- | --- |
| Age at inclusion, *median (Q1, Q3)* | 42.6 (28.3, 54,2) | 47.3 (30.0, 70.1) | 38.0 (26.2, 60.8) | 0.423 | 0.832, 0.384 |
| Sex, female, *n (%)* | 29 (52.7) | 24 (48.0) | 52 (51.0) | 0.628 | 0.834, 0.863 |
| Charlson Comorbidity Index, *n (%)*  0  1-2  >2 | 29 (52.7)  24 (43.6)  2 (3.6) | 30 (60.0)  12 (24.0)  8 (16.0) | 55 (53.9)  32 (31.4)  15 (14.7) | **0.028*** | 0.057, 0.658 |
| BMI (kg/m^2^), *mean (SD)* | 26.4 (4.7) | 25.1 (3.3) | 24.4 (3.9) | 0.105 | **0.005*,** 0.296 |
| BMI, *n (%)*  Underweight (<18.5 kg/m^2^)  Normal weight (18.5-24.9 kg/m^2^)  Overweight (≥25.0 kg/m^2^)  Obesity (≥30.0 kg/m^2^) | 1 (1.8)  24 (43.6)  19 (34.6)  11 (20.0) | 1 (2.0)  25 (50.0)  20 (40.0)  4 (8.0) | 1 (1.0)  60 (58.8)  32 (31.4)  9 (8.8) | 0.333 | 0.104, 0.608 |
| Malnutrition, *n (%)* | 1 (1.8) | 5 (10.0) | 0 (0.0) | 0.100 | 0.350, **0.003*** |
| Education level, *n (%)* ^a^  Primary education  Secondary education  Intermediate vocational education Higher vocational education University | 0 (0.0)  17 ( 30.9)  24 (43.6)  12 (21.8)  2 (3.6) | 0 (0.0)  8 (16.0)  14 (28.0)  13 (26.0)  15 (30.0) | 0 (0.0)  16 (16.0)  15 (15.0)  26 (26.0)  43 (43.0) | **0.001*** | **<0.001***, 0.134 |
| Employment status, *n (%)* ^a^  Working full-time  Working part-time  Studying  Retired  Sick leave  Partially or fully unfit to work  Not working for other reasons | 14 (25.5)  15 (27.3)  9 (16.4)  6 (10.9)  3 (5.5)  8 (14.6)  0 (0.0) | 21 (42.0)  12 (24.0)  4 (8.0)  7 (14.0)  3 (6.0)  1 (2.0)  2 (4.0) | 53 (53.0)  14 (14.0)  16 (16.0)  14 (14.0)  1 (1.0)  1 (1.0)  1 (1.0) | 0.083 | **<0.001***, 0.120 |
| Smoking status, *n (%)*  Current smoker  Former smoker  Quit <6 months ago ^b^  Quit ≥6 months ago ^b^ Never smoked | 7 (12.7)  21 (38.2)  19 (95.0)  1 (5.0)  27 (49.1) | 5 (10.0)  20 (40.0)  0 (0)  20 (0)  25 (50.0) | 11 (10.8)  16 (15.7)  0 (0)  16 (15.7)  75 (73.5) | 0.906 | ***<*0.004***, ***<*0.004*** |
| CIS total score, *median (Q1, Q3)* ^c^  CIS subjective fatigue, *median (Q1, Q3)* ^c^  CIS concentration, *median (Q1, Q3)* ^c^  CIS motivation, *median (Q1, Q3)*  CIS activity, *median (Q1, Q3)* | 65 (47, 85)  32 (23, 42)  15 (9, 22)  10 (8, 14)  7 (5, 11) | 56 (42, 80)  27 (18, 38)  13 (8, 19)  9 (7, 13)  7 (5, 11) | 43 (32, 59)  16 (12, 24)  11 (8, 16)  8 (5, 11)  6 (4, 9) | 0.214  0.054  0.416  0.444  0.933 | **<0.001***, **<0.001***  **<0.001***, **<0.001***  0.074, 0.427  **0.002***, **0.023***  0.136, 0.090 |
| IPAQ total MET-min/week, *median (Q1, Q3)* ^d^  IPAQ walking MET-min/week, *median (Q1, Q3)* ^e^  IPAQ moderate intensity MET-min/week, *median (Q1, Q3)* ^f^  IPAQ vigorous intensity MET-min/week, *median (Q1, Q3)* ^g^  IPAQ sitting min/day, *median (Q1, Q3) ^h^* | 3690 (2376, 6045)  1386 (730, 2772)  720 (240, 1740)  720 (0, 2520)  300 (180, 480) | 2853 (2259, 7428)  924 (495, 1386)  880 (370, 1920)  480 (0, 1920)  480 (300, 540) | 3639 (2346, 6375)  990 (446, 1386)  960 (480, 2160)  1440 (480, 2400)  360 (240, 540) | 0.521  **0.008***  0.189  0.545  0.053 | 0.854, 0.473  **0.007***, 0.718  0.119, 0.908  **0.020***, **0.005***  0.189, 0.417 |
| Disease specific characteristics |  |  |  |  |  |
| Montreal age at diagnosis, *n (%)*  A1: ≤ 16 years  A2: 17-40 years  A3: > 40 years | 5 (9.1)  38 (69.1)  12 (21.8) | 2 (4.0)  37 (74.0)  11 (22.0) |  | 0.601 |  |
| Montreal disease location [CD], *n (%)*  L1: ileal  L2: colonic  L3: ileocolonic  + Perianal disease  + Upper gastrointestinal disease | 18 (32.7)  12 (21.8)  25 (45.5)  10 (18.2)  5 (9.1) |  |  |  |  |
| Montreal disease behaviour [CD], *n (%)*  B1: non-stricturing, non-penetrating  B2: stricturing  B3: penetrating | 33 (60.0)  9 (16.4)  13 (23.6) |  |  |  |  |
| Montreal disease extension [UC], *n (%)*  E1: proctitis  E2: left-sided colitis  E3: pancolitis |  | 5 (10.0)  17 (34.0)  28 (56.0) |  |  |  |
| Disease duration (years), *median (Q1, Q3)* | 10.8 (6.0, 21.0) | 10.4 (4.9, 21.0) |  | 0.873 |  |
| HBI score, *median (Q1, Q3)* | 3 (2, 5) |  |  |  |  |
| SCCAI score, *median (Q1, Q3)* |  | 1 (0, 2) |  |  |  |
| Clinical disease activity, *n (%)* ^i^  Remission  Mild disease activity  Moderate disease activity | 40 (72.7)  14 (25.5)  1 (1.8) | 40 (80.0)  10 (20.0)  0 (0.0) |  | 0.566 |  |
| Faecal calprotectin (μg/g), *median (Q1, Q3)* ^j^ | 56 (25, 212) | 32 (15, 199) |  | 0.352 |  |
| Biochemical disease activity, *n (%)*  ^j^  Remission  Active disease | 43 (82.7)  9 (17.3) | 37 (80.4)  9 (19.6) |  | 0.773 |  |
| IBD-Control-8 score, *median (Q1, Q3)* ^k^ | 13 (9, 16) | 16 (13, 16) |  |  |  |
| IBD-Control-VAS score, *median (Q1, Q3)* ^k^ | 80 (66, 90) | 95 (81, 100) |  |  |  |
| EIM during disease course, *n (%)* ^l^  ≥1 EIM during disease course  Uveitis or scleritis  Primary sclerosing cholangitis  Arthralgia  Arthritis  Axial  Peripheral | 35 (63.6)  7 (12.7)  1 (1.8)  21 (38.2)  12 (21.8)  5 (9.1)  8 (14.6) | 17 (34.0)  1 (2.0)  1 (2.0)  10 (20.0)  6 (12.0)  2 (4.0)  5 (10.0) |  | **0.002***  0.062  1.000  0.054  0.205  0.441  0.562 |  |
| Current IBD medication*, n (%)*  None  Mesalazine only  (Topical) corticosteroids  Immunomodulators  Biologic agents | 11 (20.0)  2 (3.6)  4 (7.3)  9 (16.4)  33 (60.0) | 8 (16.0)  16 (32.0)  3 (6.0)  4 (8.0)  22 (44.0) |  | **0.009*** |  |
| Number of biologicals during disease course, *median (Q1, Q3)* | 1 (0, 3) | 1 (0, 2) |  | 0.333 |  |
| Prior intestinal resection, *n (%)* | 20 (36.4) | 2 (4.0) |  | **<0.001*** |  |

Abbreviations: BMI = body mass index, CD = Crohn’s disease, CIS = checklist individual strength, EIM = extraintestinal manifestations, HBI = Harvey Bradshaw index, HC = healthy controls, IBD = inflammatory bowel disease, IPAQ = international physical activity questionnaire, MET = metabolic equivalent of task, n = number of patients, Q1 = 1st quartile, Q3 = 3rd quartile, SCCAI = simple clinical colitis activity index, SD = standard deviation, UC = ulcerative colitis, VAS = visual analogue scale. * Significant *p* < 0.05

^a^ *n =* 2 missing in the HC group

^b^ *n =* 1 missing in the CD group

^c^ *n =* 1 missing in the HC group

^c^ available in *n =* 49 in the CD group, *n =* 43 in the UC group, and *n =* 91 in the HC group, missing data due to responses of 'I don't know'

^e^ available in *n =* 50 in the CD group , *n =* 46 in the UC group, and *n =* 98 in the HC group, missing data due to responses of 'I don't know'

^f^ available in *n =* 54 in the CD group, *n =* 48 in the UC group, and *n =* 99 in the HC group, missing data due to responses of 'I don't know'

^g^ available in *n =* 54 in the CD group, *n =* 47 in the UC group, and *n =* 94 in the HC group, missing data due to responses of 'I don't know'

^h^ available in *n =* 52 in the CD group, *n =* 46 in the UC group and *n =* 97 in the HC group, missing data due to responses of 'I don't know'

^i^ clinical disease activity according to the HBI for CD and the SCCAI for UC; remission was defined as HBI < 5 or SCCAI < 3, mild disease activity as HBI 5-7 or SCCAI 3-5, and moderate disease activity as HBI 8-16 or SCCAI 6-11

^j^ = available in *n =* 52 in the CD group and *n =* 46 in the UC group; remission was defined as faecal calprotectin < 250 ug/g

^k^ = available in *n =* 31 in the CD group and *n =* 25 in the UC group within 4 months around inclusion

^l^ = values represent the number (%) of unique patients who experienced EIM at least once during their disease course. Recurrent episodes of the same EIM in the same individual were not counted multiple times. A single patient may have had more than one type of EIM.

**Supplementary Table 2.** Multivariable linear regression analysis of the associations of demographic and clinical characteristics with physical fitness test outcomes for body composition, cardiorespiratory fitness, muscular strength, and flexibility in patients with IBD and healthy controls.

| Variable | Four-site skinfold thickness | | Steep ramp test | | 60-second sit-to-stand test | | Hand-held dynamometry hamstring strength | | Sit-and-reach test | |
| --- | --- | --- | --- | --- | --- | --- | --- | --- | --- | --- |
|  | **B (95% CI)** | ***p*** | **B (95% CI)** | ***p*** | **B (95% CI)** | ***p*** | **B (95% CI)** | ***p*** | **B (95% CI)** | ***p*** |
| IBD present | 1.71 (0.22, 3.41) | **0.047*** | -0.38 (-0.59, -0.16) | ***<*0.001*** | -3.56 (-6.61, -0.51) | **0.023*** | -0.16 (-0.32, -0.01) | **0.039*** | -0.49 (-3.35, 2.37) | 0.737 |
| Sex, male | -8.94 (-10.47, -7.41) | ***<*0.001*** | -0.55 (0.35, 0.74) | ***<*0.001*** | 2.55 (-0.21, 5.31) | 0.070 | 0.34 (0.20, 0.09) | ***<*0.001*** | -8.34 (-10.93, -5.76) | **<0.001*** |
| Age (years) | 0.28 (0.19, 0.38) | ***<*0.001*** | -0.04 (-0.05, -0.02) | ***<*0.001*** | -0.15 (-0.32, 0.02) | 0.084 | -0.01 (-0.02, 0.00) | **0.004*** | -0.05 (-0.21, 0.11) | 0.541 |
| CCI | -0.81 (-2.16, 0.55) | 0.240 | 0.02 (-0.16, 0.19) | 0.851 | -2.00 (-4.44, 0.44) | 0.108 | -0.04 (-0.16, 0.09) | 0.582 | -0.16 (-2.45, 2.12) | 0.888 |
| Smoking status  Current smoker  Former smoker  Never smoked | 0.46 (-2.05, 2.97)  0.67 (-1.27, 2.61)  REF | 0.717  0.496  REF | -0.44 (-0.76, -0.13)  -0.37 (-0.61, -0.12)  REF | **0.007***  **0.004***  REF | -4.28 (-7.26, 1.80)  -4.28 (-7.78, -0.78)  REF | 0.236  **0.017***  REF | -0.07 (-0.30, 0.16)  -0.08 (-0.26, 0.10)  REF | 0.573  0.369  REF | 1.25 (-3.00, 5.49)  -1.02 (-4.31, 2.26)  REF | 0.562  0.540  REF |
| Educational level ^a^  Low/medium  ^a^  High ^b^ | REF  -1.33 (-2.97, 0.31) | REF  0.111 | REF  0.36 (0.15, 0.57) | REF  ***<*0.001*** | REF  2.96 (0.00, 5.92) | REF  0.050 | REF  0.04 (-0.11, 0.19) | REF  0.563 | REF  0.66 (-2.11, 3.44) | REF  0.637 |

Each column represents a separate multivariable linear regression model including all listed variables as covariates. Abbreviations: CCI = Charlson comorbidity index, CI = confidence interval, IBD = inflammatory bowel disease. * Significant *p* < 0.05
^a^ low and medium educational level, including primary education, secondary education, and intermediate vocational education

^b^ high educational level, including higher vocational education and university

**Supplementary Table 3.** Multivariable linear regression analysis of the associations of demographic and clinical characteristics with physical fitness test outcomes for muscular endurance in patients with IBD and healthy controls.

| Variable | Quadriceps peak torque fatigue index | | Hamstring peak torque fatigue index | | Quadriceps work fatigue index | | Hamstring work fatigue index | |
| --- | --- | --- | --- | --- | --- | --- | --- | --- |
|  | **B (95% CI)** | ***p*** | **B (95% CI)** | ***p*** | **B (95% CI)** | ***p*** | **B (95% CI)** | ***p*** |
| IBD present | 0.33 (-2.57, 3.23) | 0.821 | 0.15 (-3.12, 3.42) | 0.927 | -0.46 (-4.88, 3.97) | 0.840 | -0.47 (-4.32, 3.38) | 0.810 |
| Sex, male | -0.96 (-3.59, 1.66) | 0.470 | 2.10 (-0.86, 5.06) | 0.164 | -2.83 (-6.83, 1.18) | 0.166 | -0.53 (-4.01, 2.95) | 0.765 |
| Age (years) | -0.11 (-0.27, 0.05) | 0.163 | -0.06 (-0.24, 0.12) | 0.544 | -0.18 (-0.43, 0.06) | 0.140 | -0.06 (-0.27, 0.16) | 0.611 |
| CCI | -0.15 (-3.47, 1.17) | 0.329 | -3.23 (-5.84, -6.62) | **0.016*** | -1.13 (-4.67, 2.42) | 0.532 | -4.66 (-7.74, -1.59) | **0.003*** |
| Smoking status  Current smoker  Former smoker  Never smoked | 4.73 (0.42, 9.03)  2.07 (-1.26, 5.39)  REF | **0.032***  0.222  REF | 5.64 (0.78, 10.49)  2.95 (-0.80, 6.70)  REF | **0.023***  0.123  REF | 7.17 (0.60, 13.74)  4.45 (-0.64, 9.53)  REF | **0.033***  0.086  REF | 3.74 (-1.97, 9.45)  3.48 (0.93, 7.90)  REF | 0.198  0.122  REF |
| Educational level ^a^  Low/medium  ^a^  High ^b^ | REF  -2.19 (-5.00, 0.62) | REF  0.126 | REF  2.87 (-0.30, 6.04) | REF  0.076 | REF  -3.48 (-7.77, 0.82) | REF  0.112 | REF  3.53 (-0.20, 7.26) | REF  0.064 |

Each column represents a separate multivariable linear regression model including all listed variables as covariates. Abbreviations: CCI = Charlson comorbidity index, CI = confidence interval, IBD = inflammatory bowel disease. * Significant *p* < 0.05
^a^ low and medium educational level, including primary education, secondary education, and intermediate vocational education

^b^ high educational level, including higher vocational education and university

**Supplementary Table 4.** Sensitivity analysis: Multivariable linear regression analysis of the associations of demographic and clinical characteristics with physical fitness test outcomes for body composition, cardiorespiratory fitness, muscular strength, and flexibility in patients with IBD in biochemical remission (n=80) and healthy controls.

| Variable | Four-site skinfold thickness | | Steep ramp test | | 60-second sit-to-stand test | | Hand-held dynamometry hamstring strength | | Sit-and-reach test | |
| --- | --- | --- | --- | --- | --- | --- | --- | --- | --- | --- |
|  | **B (95% CI)** | ***p*** | **B (95% CI)** | ***p*** | **B (95% CI)** | ***p*** | **B (95% CI)** | ***p*** | **B (95% CI)** | ***p*** |
| IBD present | 1.92 (0.15, 3.70) | **0.034*** | -0.40 (-0.64, -0.17) | ***<*0.001*** | -4.30 (-7.37, -1.22) | **0.006*** | -0.21 (-0.37, -0.04) | **0.015*** | 0.26 (-2.75, 3.28) | 0.864 |
| Sex, male | -9.20 (-10.80, -7.60) | ***<*0.001*** | -0.55 (0.34, 0.76) | ***<*0.001*** | 1.67 (-1.09, 4.44) | 0.234 | 0.30 (0.15, 0.45) | ***<*0.001*** | -8.06 (-10.77, -5.35) | **<0.001*** |
| Age (years) | 0.27 (0.18, 0.37) | ***<*0.001*** | -0.03 (-0.05, -0.02) | ***<*0.001*** | -0.09 (-0.26, 0.07) | 0.280 | -0.01 (-0.02, 0.00) | **0.012*** | -0.04 (-0.20, 0.12) | 0.626 |
| CCI | -0.62 (-2.00, 0.76) | 0.375 | 0.00 (-0.18, 0.18) | 0.973 | -2.52 (-4.91, -0.13) | **0.039*** | -0.05 (-0.18, 0.08) | 0.436 | 0.07 (-2.27, 2.41) | 0.953 |
| Smoking status  Current smoker  Former smoker  Never smoked | 0.20 (-2.36, 2.77)  0.51 (-1.53, 2.55)  REF | 0.875  0.624  REF | -0.44 (-0.77, -0.10)  -0.35 (-0.62, -0.08)  REF | **0.011***  **0.010***  REF | -2.51 (-6.94, 1.93)  -3.25 (-6.78, 0.28)  REF | 0.266  0.071  REF | -0.03 (-0.27, 0.21)  -0.04 (-0.23, 0.15)  REF | 0.805  0.654  REF | 0.85 (-3.51, 5.20)  -1.64 (-5.10, 1.83)  REF | 0.702  0.353  REF |
| Educational level ^a^  Low/medium  ^a^  High ^b^ | REF  -1.12 (-2.83, 0.58) | REF  0.195 | REF  0.33 (0.11, 0.56) | REF  **0.004*** | REF  3.27 (0.31, 6.22) | REF  **0.030*** | REF  0.06 (-0.10, 0.22) | REF  0.455 | REF  0.60 (-2.29, 3.50) | REF  0.681 |

Each column represents a separate multivariable linear regression model including all listed variables as covariates. Abbreviations: CCI = Charlson comorbidity index, CI = confidence interval, IBD = inflammatory bowel disease. * Significant *p* < 0.05
^a^ low and medium educational level, including primary education, secondary education, and intermediate vocational education

^b^ high educational level, including higher vocational education and university

**Supplementary Table 5.** Sensitivity analysis: Multivariable linear regression analysis of the associations of demographic and clinical characteristics with physical fitness test outcomes for muscular endurance in patients with IBD in biochemical remission (n=80) and healthy controls.

| Variable | Quadriceps peak torque fatigue index | | Hamstring peak torque fatigue index | | Quadriceps work fatigue index | | Hamstring work fatigue index | |
| --- | --- | --- | --- | --- | --- | --- | --- | --- |
|  | **B (95% CI)** | ***p*** | **B (95% CI)** | ***p*** | **B (95% CI)** | ***p*** | **B (95% CI)** | ***p*** |
| IBD present | -0.50 (-3.50, 2.50) | 0.741 | -0.20 (-3.66, 3.25) | 0.908 | -0.40 (-4.74, 3.95) | 0.857 | -1.14 (-5.19, 1.91) | 0.579 |
| Sex, male | -1.20 (-3.90, 1.50) | 0.382 | 2.13 (-0.98, 5.25) | 0.178 | -2.61 (-6.17, 1.65) | 0.255 | -0.38 (-4.02, 3.26) | 0.837 |
| Age (years) | -0.08 (-0.24, 0.08) | 0.311 | -0.01 (-0.19, 0.18) | 0.956 | -0.10 (-0.34, 0.13) | 0.377 | 0.00 (-0.22, 0.22) | 1.000 |
| CCI | -1.50 (-3.83, 0.83) | 0.205 | -3.38 (-6.07, -0.69) | **0.014*** | -1.56 (-4.93, 1.82) | 0.363 | -5.09 (-8.24, -1.94) | **0.002*** |
| Smoking status  Current smoker  Former smoker  Never smoked | 4.83 (0.50, 9.16)  2.10 (-1.35, 5.55)  REF | **0.029***  0.231  REF | 5.39 (0.40, 10.38)  2.55 (-1.42, 6.53)  REF | **0.035***  0.207  REF | 7.24 (0.97, 13.50)  3.46 (-1.53, 8.45)  REF | **0.024***  0.173  REF | 3.28 (-2.57, 9.12)  3.36 (-1.29, 8.02)  REF | 0.270  0.156  REF |
| Educational level ^a^  Low/medium  ^a^  High ^b^ | REF  -1.58 (-4.46, 1.31) | REF  0.282 | REF  3.28 (-0.05, 6.60) | REF  0.053 | REF  -2.13 (-6.30, 2.05) | REF  0.316 | REF  3.67 (-0.22, 7.57) | REF  0.064 |

Each column represents a separate multivariable linear regression model including all listed variables as covariates. Abbreviations: CCI = Charlson comorbidity index, CI = confidence interval, IBD = inflammatory bowel disease. * Significant *p* < 0.05
^a^ low and medium educational level, including primary education, secondary education, and intermediate vocational education

^b^ high educational level, including higher vocational education and university

**Supplementary Table 6.** Physical fitness outcomes of patients with IBD (*n* = 105), separately for patients with CD (*n* = 55) and UC (*n* = 50), and healthy controls (*n* = 102).

| Physical fitness outcomes | IBD  (*n* = 105) | CD  (*n* = 55) | UC  (*n* = 50) | HC  (*n* = 102) | *p*  (CD *vs* UC) | *p*  (CD *vs* HC,  UC *vs* HC) |
| --- | --- | --- | --- | --- | --- | --- |
| Body fat percentage, *mean (SD)* | 29.5 (8.7) | 30.2 (8.7) | 28.7 (8.7) | 26.9 (7.6) | 0.369 | **0.014*,** 0.193 |
| Steep ramp test WR_peak_ (W/kg), *mean (SD)* | 4.2 (1.0) | 4.2 (1.1) | 4.3 (1.0) | 4.8 (1.0) | 0.404 | **<0.001*, 0.004*** |
| 60-second sit-to-stand test (repetitions), *mean (SD)* | 42 (12) | 41 (13) | 42 (11) | 47 (10) | 0.840 | **0.007*, 0.011*** |
| Hand-held dynamometry hamstring strength (N/kg), *mean (SD)* | 3.0 (0.65) | 3.0 (0.66) | 3.0 (0.64) | 3.2 (0.51) | 0.968 | **0.046***, 0.055 |
| Quadriceps peak torque fatigue index, *median (Q1, Q3)* | 32.9 (25.6, 40.5) | 35.9 (27.6, 42.8) | 30.0 (23.4, 35,1) | 31.0 (24.1, 37.1) | **<0.001*** | **0.004*,** 0.319 |
| Hamstring peak torque fatigue index, median *(Q1, Q3)* | 27.6 (18.1, 35.7) | 27.6 (22.7, 37.1) | 27.0 (17.8, 35.2) | 27.0 (19.7, 34.6) | 0.434 | 0.416, 0.785 |
| Quadriceps work fatigue index, *median (Q1, Q3)* | 37.9 (28.7, 44.4) | 42.2 (32.9, 48.3) | 33.5 (25.2, 41.5) | 35.1 (27.2, 41.3) | **<0.001*** | **<0.001*,** 0.435 |
| Hamstring work fatigue index, median *(Q1, Q3)* | 35.4 (28.1, 44.1) | 36.4 (29.6 45.1) | 33.6 (25.0, 41.8) | 37.7 (27.4, 44.5) | 0.162 | 0.732, 0.255 |
| Sit-and-reach distance (cm), *mean (SD)* | 25 (10) | 24 (10) | 25 (10) | 26 (10) | 0.667 | 0.329, 0.657 |

Abbreviations: CD = Crohn's disease, HC = healthy controls, IBD = inflammatory bowel disease, Q1 = 1st quartile, Q3 = 3rd quartile, SD = standard deviation, UC = ulcerative colitis, WR_peak_ = work rate at peak exercise. * Significant *p <*0.05

**Supplementary Table 7.** Multivariable linear regression analysis of the associations of demographic and clinical characteristics with physical fitness test outcomes for body composition, cardiorespiratory fitness, muscular strength, and flexibility in patients with CD and healthy controls.

| Variable | Four-site skinfold thickness | | Steep ramp test | | 60-second sit-to-stand test | | Hand-held dynamometry hamstring strength | | Sit-and-reach test | |
| --- | --- | --- | --- | --- | --- | --- | --- | --- | --- | --- |
|  | **B (95% CI)** | ***p*** | **B (95% CI)** | ***p*** | **B (95% CI)** | ***p*** | **B (95% CI)** | ***p*** | **B (95% CI)** | ***p*** |
| CD present | 2.65 (0.58, 4.72) | **0.012*** | -0.40 (-0.67, -0.13) | **0.004*** | -2.80 (-6.70, 1.10) | 0.157 | -0.14 (-0.33, 0.05) | 0.155 | 1.08 (-4.64, 2.49) | 0.551 |
| Sex, male | -8.83 (-10.57, -7.10) | **<0.001*** | 0.47 (0.25, 0.70) | **<0.001*** | -1.86 (-1.41, 5.14) | 0.262 | 0.31 (0.15, 0.46) | **<0.001*** | -8.18 (-11.17, -5.18) | **<0.001** |
| Age (years) | 0.22 (0.12, 0.33) | **<0.001*** | -0.04 (-0.05, -0.02) | **<0.001*** | -0.13 (-0.33, 0.07) | 0.189 | 0.01 (-0.02, 0.00) | **0.042*** | 0.00 (-0.19, 0.18) | 0.973 |
| CCI | -0.07 (-1.65, 1.51) | 0.933 | 0.03 (-0.18, 0.24) | 0.759 | -1.79 (-4.77, 1.18) | 0.235 | -0.05 (-0.19, 0.10) | 0.506 | -0.58 (-3.30, 2.14) | 0.674 |
| Smoking status  Current smoker  Former smoker  Never smoked | -0.39 (-3.22, 2.48)  1.25 (-1.07, 3.56)  REF | 0.787  0.288  REF | -0.38 (-0.75, -0.01)  -0.48 (-0.78, -0.17)  REF | **0.047***  **0.002***  REF | -2.23 (-7.58, 3.11)  -4.98 (-9.34, 0.61)  REF | 0.411  **0.026***  REF | 0.00 (-0.26, 0.26)  -0.17 (-0.38, 0.04)  REF | 0.984  0.116  REF | 1.51 (-3.38, 6.40)  -2.79 (-6.78, 1.20)  REF | 0.543  0.169  REF |
| Educational level ^a^  Low/medium  ^a^  High ^b^ | REF  -0.86 (-2.81, 1.09) | REF  0.385 | REF  0.38 (0.13, 0.64) | REF  **0.004*** | REF  4.25 (0.58, 7.92) | REF  **0.024*** | REF  0.08 (-0.10, 0.26) | REF  0.374 | REF  -0.08 (-3.44, 3.28) | REF  0.963 |

Abbreviations: CCI = Charlson comorbidity index, CD = Crohn’s disease, CI = confidence interval. * Significant *p* < 0.05
^a^ low and medium educational level, including primary education, secondary education, and intermediate vocational education

^b^ high educational level, including higher vocational education and university

**Supplementary Table 8.** Multivariable linear regression analysis of the associations of demographic and clinical characteristics with physical fitness test outcomes for muscular endurance in patients with CD and healthy controls.

| Variable | Quadriceps peak torque fatigue index | | Hamstring peak torque fatigue index | | Quadriceps work fatigue index | | Hamstring work fatigue index | |
| --- | --- | --- | --- | --- | --- | --- | --- | --- |
|  | **B (95% CI)** | ***p*** | **B (95% CI)** | ***p*** | **B (95% CI)** | ***p*** | **B (95% CI)** | ***p*** |
| CD present | 3.97 (0.51, 7.43) | **0.025*** | 1.85 (-2.24, 5.95) | 0.373 | 4.56 (-0.33, 9.45) | 0.067 | 1.58 (-3.21, 6.37) | 0.516 |
| Sex, male | -0.74 (-3.65, 2.17) | 0.616 | 2.76 (-0.68, 6.20) | 0.115 | -0.77 (-4.88, 3.33) | 0.710 | 0.61 (-3.41, 4.63) | 0.765 |
| Age (years) | 0.01 (-0.17, 0.18) | 0.948 | 0.02 (-0.20, 0.23) | 0.833 | 0.03 (-0.22, 0.28) | 0.804 | 0.08 (-0.17, 0.33) | 0.526 |
| CCI | -2.63 (-5.28, 0.01) | 0.051 | -4.22 (-7.35, -1.09) | **0.009*** | -3.56 (-7.30, 0.17) | 0.061 | -6.94 (-10.60, -3.29) | **<0.001*** |
| Smoking status  Current smoker  Former smoker  Never smoked | 7.44 (2.69, 12.19)  0.68 (-3.20, 4.56)  REF | **0.002***  0.731  REF | 7.10 (1.48, 12.72)  4.00 (-0.59, 8.59)  REF | **0.014***  0.087  REF | 9.07 (2.36, 15.78)  1.24 (-4.24, 6.71)  REF | **0.008***  0.656  REF | 7.43 (0.86, 14.00)  3.98 (-1.38, 9.35)  REF | **0.027***  0.144  REF |
| Educational level ^a^  Low/medium  ^a^  High ^b^ | REF  -0.05 (-3.31, 3.22) | REF  0.978 | REF  4.70 (0.84, 8.56) | REF  **0.017*** | REF  -0.55 (-5.16, 4.06) | REF  0.814 | REF  5.66 (1.15, 10.18) | REF  **0.014*** |

Abbreviations: CCI = Charlson comorbidity index, CD = Crohn’s disease, CI = confidence interval. * Significant *p* < 0.05
^a^ low and medium educational level, including primary education, secondary education, and intermediate vocational education

^b^ high educational level, including higher vocational education and university

**Supplementary Table 9.** Multivariable linear regression analysis of the associations of demographic and clinical characteristics with physical fitness test outcomes for body composition, cardiorespiratory fitness, muscular strength, and flexibility in patients with UC and healthy controls.

| Variable | Four-site skinfold thickness | | Steep ramp test | | 60-second sit-to-stand test | | Hand-held dynamometry hamstring strength | | Sit-and-reach test | |
| --- | --- | --- | --- | --- | --- | --- | --- | --- | --- | --- |
|  | **B (95% CI)** | ***p*** | **B (95% CI)** | ***p*** | **B (95% CI)** | ***p*** | **B (95% CI)** | ***p*** | **B (95% CI)** | ***p*** |
| UC present | 0.72 (-1.31, 2.74) | 0.487 | -0.33 (-0.59, -0.08) | **0.011*** | -3.82 (-7.30, -0.35) | **0.031** | -0.15 (-0.33, 0.03) | 0.097 | -0.06 (-3.49, 3.36) | 0.971 |
| Sex, male | -8.14 (-9.95, -6.32) | **<0.001*** | 0.46 (0.23, 0.69) | **<0.001*** | -1.43 (-1.68, 4.55) | 0.365 | 0.26 (0.10, 0.42) | **0.002*** | -8.40 (-11.47, -5.33) | **<0.001*** |
| Age (years) | 0.30 (0.20, 0.41) | **<0.001*** | -0.03 (-0.05, -0.02) | **<0.001*** | -0.07 (-0.25, 0.11) | 0.462 | -0.01 (-0.02, 0.00) | **0.030*** | -0.06 (-0.23, 0.12) | 0.532 |
| CCI | -1.25 (-2.76, 0.27) | 0.107 | 0.00 (-0.19, 0.19) | 0.987 | -2.95 (-5.55, -0.34) | **0.027*** | -0.05 (-0.19, 0.08) | 0.458 | -0.21 (-2.77, 2.35) | 0.872 |
| Smoking status  Current smoker  Former smoker  Never smoked | 0.03 (-2.96, 3.01)  0.72 (-1.60, 3.03)  REF | 0.987  0.543  REF | -0.39 (-0.76, -0.01)  -0.27 (-0.56, 0.02)  REF | **0.043***  0.064  REF | -2.11 (-7.24, 3.02)  -4.17 (-8.14, -0.19)  REF | 0.417  **0.040***  REF | -0.01 (-0.27, 0.26)  -0.09 (-0.29, 0.12)  REF | 0.956  0.412  REF | 0.23 (-4.82, 5.27)  0.45 (-3.46, 4.35)  REF | 0.930  0.822  REF |
| Educational level ^a^  Low/medium  ^a^  High ^b^ | REF  -1.67 (-3.58, 0.24) | REF  0.086 | REF  0.39 (0.15, 0.63) | REF  **0.002*** | REF  3.16 (-0.12, 6.44) | REF  0.059 | REF  0.08 (-0.09, 0.25) | REF  0.359 | REF  1.60 (-1.62, 4.83) | REF  0.328 |

Abbreviations: CCI = Charlson comorbidity index, CI = confidence interval, UC = ulcerative colitis. * Significant *p* < 0.05
^a^ low and medium educational level, including primary education, secondary education, and intermediate vocational education

^b^ high educational level, including higher vocational education and university

**Supplementary Table 10.** Multivariable linear regression analysis of the associations of demographic and clinical characteristics with physical fitness test outcomes for muscular endurance in patients with UC and healthy controls.

| Variable | Quadriceps peak torque fatigue index | | Hamstring peak torque fatigue index | | Quadriceps work fatigue index | | Hamstring work fatigue index | |
| --- | --- | --- | --- | --- | --- | --- | --- | --- |
|  | **B (95% CI)** | ***p*** | **B (95% CI)** | ***p*** | **B (95% CI)** | ***p*** | **B (95% CI)** | ***p*** |
| UC present | -2.18 (-5.54, 1.18) | 0.202 | -1.08 (-4.81, 2.66) | 0.569 | -4.14 (-9.58, 1.31) | 0.136 | -2.34 (-6.86, 2.17) | 0.307 |
| Sex, male | -1.33 (-4.34, 1.69) | 0.385 | 3.51 (0.17, 6.86) | **0.040*** | -4.03 (-8.91, 0.86) | 0.105 | 1.00 (-3.05, 5.04) | 0.627 |
| Age (years) | -0.16 (-0.33, 0.02) | 0.080 | -0.04 (-0.24, 0.15) | 0.673 | -0.24 (-0.52, 0.04) | 0.096 | -0.06 (-0.30, 0.17) | 0.595 |
| CCI | -0.43 (-2.95, 2.09) | 0.736 | -3.58 (-6.38, -0.79) | **0.012*** | -0.07 (-4.15, 4.00) | 0.972 | -4.68 (-8.06, -1.30) | **0.007*** |
| Smoking status  Current smoker  Former smoker  Never smoked | 3.10 (-1.86, 8.05)  1.25 (-2.59, 5.08)  REF | 0.219  0.522  REF | 6.34 (0.84, 11.85)  1.53 (-2.73, 5.80)  REF | **0.024***  0.479  REF | 5.99 (-2.03, 14.02)  5.41 (-0.81, 11.63)  REF | 0.142  0.088  REF | 3.10 (-3.56, 9.76)  2.81 (-2.35, 7.96)  REF | 0.359  0.284  REF |
| Educational level ^a^  Low/medium  ^a^  High ^b^ | REF  -1.94 (-5.11, 1.23) | REF  0.228 | REF  3.21 (-0.31, 6.73) | REF  0.074 | REF  -3.49 (-8.62, 1.65) | REF  0.182 | REF  3.01 (-1.24, 7.27) | REF  0.164 |

Abbreviations: CCI = Charlson comorbidity index, CI = confidence interval, UC = ulcerative colitis. * Significant *p* < 0.05
^a^ low and medium educational level, including primary education, secondary education, and intermediate vocational education

^b^ high educational level, including higher vocational education and university
